# The national and subnational prevalence of cataract and cataract blindness in China: a systematic review and meta-analysis

**DOI:** 10.7189/jogh.08-010804

**Published:** 2018-06

**Authors:** Peige Song, He Wang, Evropi Theodoratou, Kit Yee Chan, Igor Rudan

**Affiliations:** 1 Centre for Global Health Research, Usher Institute of Population Health Sciences and Informatics, University of Edinburgh, Edinburgh, Scotland, United Kingdom; 2 The First Affiliated Hospital of Zhengzhou University, Zhengzhou, Henan, China

## Abstract

**Background:**

Cataract is the second leading cause of visual impairment and the first of blindness globally. However, for the most populous country, China, much remains to be understood about the scale of cataract and cataract blindness. We aimed to investigate the prevalence of cataract and cataract blindness in China at both the national and subnational levels, with projections till 2050.

**Methods:**

In this systematic review and meta-analysis, China National Knowledge Infrastructure (CNKI), Wanfang, Chinese Biomedicine Literature Database (CBM-SinoMed), PubMed, Embase, and Medline were searched using a comprehensive search strategy to identify all relevant articles on the prevalence of cataract or cataract blindness in Chinese population published from January 1990 onwards. We fitted a multilevel mixed-effects meta-regression model to estimate the prevalence of cataract, and a random-effects meta-analysis model to pool the overall prevalence of cataract blindness. The United Nations Population Division (UNPD) data were used to estimate and project the number of people with cataract and cataract blindness from 1990 to 2050. According to different demographic and geographic features in the six geographic regions in China, the national numbers of people with cataract in the years 2000 and 2010 were distributed to each region.

**Results:**

In males, the prevalence of any cataract (including post-surgical cases) ranged from 6.71% (95% CI = 5.06-8.83) in people aged 45-49 years to 73.01% (95% CI = 65.78-79.2) in elderly aged 85-89 years. In females, the prevalence of any cataract increased from 8.39% (95% CI = 6.36-10.98) in individuals aged 45-49 years to 77.51% (95% CI = 71.00-82.90) in those aged 85-89 years. For age-related cataract (ARC, including post-surgical cases), in males, the prevalence rates ranged from 3.23% (95% CI = 1.51-6.80) in adults aged 45-49 years to 65.78% (95% CI = 46.72-80.82) in those aged 85-89 years. The prevalence of ARC in females was 4.72% (95% CI = 2.22-9.76) in the 45-49 years age group and 74.03% (95% CI = 56.53-86.21) in the 85–89 years age group. The pooled prevalence rate of cataract blindness (including post-surgical cases) by best corrected visual acuity (BCVA)<0.05 among middle-aged and older Chinese was 2.30% (95% CI = 1.72-3.07), and those of cataract blindness by BCVA<0.10 and cataract blindness by presenting visual acuity (PVA)<0.10 were 2.56% (95% CI = 1.94-3.38) and 4.51% (95% CI = 3.53-5.75) respectively. In people aged 45-89 years, the number of any cataract cases was 50.75 million (95% CI = 42.17-60.37) in 1990 and 111.74 million (95% CI = 92.94-132.84) in 2015, and that of ARC rose from 35.77 million (95% CI = 19.81-59.55) in 1990 to 79.04 million (95% CI = 44.14-130.85) in 2015. By 2050, it is projected that the number of people (45-89 years of age) affected by any cataract will be 240.83 million (95% CI = 206.07-277.35), and that of those with ARC will be 187.26 million (95% CI = 113.17-281.23). During 2000 and 2010, South Central China consistently owed the most cases of any cataract, whereas Northwest China the least.

**Conclusions:**

The prevalence of cataract and cataract blindness in China was unmasked. In the coming decades, cataract and cataract blindness will continue to be a leading public-health issue in China due to the ageing population. Future work should be prioritized to the promotion of high-quality epidemiological studies on cataract.

Cataract, defined as any opacity of the crystalline lens in the eye that affects clear vision, is a common condition in later life [1-3]. If left untreated, cataract can eventually progress to severe visual impairment or even blindness [4,5]. Compared to the general population, people with cataract are more likely to have substantially reduced vision-related quality of life and increased risk of comorbidity and mortality [3,6-10]. Surgery is cost-effective and successful in restoring cataract-related vision loss [11-13]. However, in many resource-deprived areas, especially remote and poor areas in developing countries, barriers in access to appropriate preventative eye care and surgical treatments still exist, presenting an enormous problem to the society in terms of social and economic burden [14-17]. Based on aetiology, cataract can be broadly classified as age-related, congenital, traumatic, secondary and drug-induced, etc., with age-related cataract (ARC) being the predominant subtype [1,3].

Despite the fact that cataract can be easily, safely and cost-efficiently treated with a standard procedure, cataract still remains the second leading cause of visual impairment and the first of blindness globally [3,5,18]. According to the latest estimates in 2010, 94 million people were visually impaired and 20 million were blind because of cataract, accounting for one third (33%) of all individuals with visual impairment and more than half (51%) of blind cases worldwide [5]. Cataract is a multifactorial disease, with ageing being the major risk factor by far [1,3]. As people live longer, the prevalence of cataract is expected to rise correspondingly, posing challenges for health systems. From the public health perspective, understanding the magnitude of cataract and cataract blindness is the first step in prevention and treatment, and can provide a basis for evidence-based policy making and public health resources allocation [19]. Despite this, comprehensive review of data regarding the prevalence and burden of cataract on a global basis has never, to the best of our knowledge, been documented previously. This may largely due to the difficulty of combining prevalence data from different national, regional, racial and ethnic groups, especially in cases where inconsistent definitions of cataract were adopted and a universally recognized standardisation was lacking.

Similarly, in the largest developing country, China, few national-representative investigations have been conducted to provide precise estimates of the prevalence of cataract or cataract blindness in general Chinese population. Nevertheless, a growing number of population-based studies on the epidemiology of cataract have been conducted across the nation during the past decades, which have reported an overall cataract prevalence of from less than 10% to more than 50% in various samples [20-23]. The substantial variations might largely come from the disparities of cataract definitions (eg, with reduced visual acuity or not, different cut-offs for defining reduced visual acuity), as well as the different characteristics of individual studies (eg, the age structure of the investigated population and the geographic location of the study sites) [21,24-26]. Until recently, no information has been compiled for the prevalence of cataract and cataract blindness in China, and the magnitude of the national number of people with cataract and cataract blindness in general Chinese population remains unclear. As Chinese population is progressively ageing, an increasing burden of cataract and cataract blindness is also expected [27-30]. If nothing else alters, the corresponding increased demand for cataract surgery will present new challenges for the Chinese health system.

In view of uncertainties about the prevalence of cataract and cataract blindness in China, we conducted a comprehensive systematic review, in both Chinese and English databases, to retrieve all population-based studies that reported the prevalence of cataract or cataract blindness in China from 1990 onwards. By using standardized definitions of cataract and cataract blindness, we quantitatively summarised the prevalence of cataract and cataract blindness in China. Furthermore, the geographic patterns and secular trend of cataract/cataract blindness prevalence were also examined to add insights to the public health domain, assisting both researchers and policymakers to prioritize health care resources needed. The principal aims of this study were: 1) to determine the prevalence and number of people affected by cataract and cataract blindness at the national level from 1990 to 2015; 2) to project the prevalence and number of people with cataract and cataract blindness at the national level till 2050; and 3) to examine regional differences in the prevalence of cataract and number of affected people from 2000 to 2010.

## METHODS

### Systematic review

This systematic review and meta-analysis conforms to the Preferred Reporting Items for Systematic reviews and Meta-Analyses (PRISMA) guidelines and the Guidelines for Accurate and Transparent Health Estimates Reporting (GATHER) statement [31,32].

#### Search strategy

Three Chinese and three English bibliographic databases, including China National Knowledge Infrastructure (CNKI), Wanfang, Chinese Biomedicine Literature Database (CBM-SinoMed), PubMed, Embase, and Medline, were searched using a comprehensive search strategy to identify all relevant articles on the prevalence of cataract or cataract blindness in general Chinese population published from January 1990 onwards. Search terms that related to cataract (“cataract” or “cataracts”), prevalence (“incidence”, “prevalence”, “morbidity”, “mortality”, “epidemiology”) and China (“China”, “Chinese”, “Hong Kong”, “Macau”, “Taiwan”) were combined in forms of controlled vocabularies (eg, Medical Subject Heading terms) and free text terms. No language restrictions were placed in the search or in the selection process. The complete search strategies are described in Table S1 in** Online Supplementary Document(Online Supplementary Document)**, which were conceived and adapted to fit features of different bibliographic databases. To supplement the database searches, reference lists of all included articles were also scrutinised to identify additional data sources.

#### Inclusion and exclusion criteria

To be included in the systematic review, studies needed to be population-based primary studies, reporting the prevalence of cataract or cataract blindness in Chinese population of a defined age and sex structure. Herein, the prevalence of cataract or cataract blindness should be calculated based on the number of affected individuals, rather than the number of affected eyes. Definitions of cataract/cataract blindness and measurements of visual acuity should be clearly stated. To avoid potentially biased information, we excluded hospital-based studies, studies that were conducted among populations of Chinese origin but residing outside China, and those confined to a specific population that was not representative of the general population (people with a specific health condition, people covered by health insurance, etc.). Narrative reviews, case reports, commentaries, editorials, conference proceedings and studies without primary data or explicit methodologies were additionally excluded. Furthermore, studies that reported prevalence rates based on self-reported data were also excluded because an underestimation was very likely. For multiple articles that reported duplicated or overlapping data of the same single study, the one with the most representative results or largest sample size was kept.

During the systematic review process, the definition of cataract was found to vary dramatically among studies (Table S2 in **Online Supplementary Document(Online Supplementary Document)**): some studies defined cataract as the presence of lens opacities without diminished visual acuity, whereas other studies documented cataract in people with reduced visual acuity, which was determined by either best corrected visual acuity (BCVA) or presenting visual acuity (PVA) [21,25,26,33]. The cut-offs of reduced visual acuity in different studies varied widely from 0.50 decimal Snellen (6/12) to 0.70 decimal Snellen (6/9) [21,26], and those for defining blindness ranged from 0.05 decimal Snellen (6/120) to 0.10 decimal Snellen (6/60) [25,33]. To ensure maximum comparability across studies and the ability to synthesize data from various studies, only studies that adopted a standardized definition of cataract or cataract blindness were eligible for the meta-analysis. Herein the standardized definitions of cataract and cataract blindness refer to the definitions that were most widely taken in Chinese epidemiological surveys (**Table 1**). In summary, cataract could be unilateral or bilateral, with reduced visual acuity (BCVA≤0.70), and include pseudophakia/aphakia. Cataract was further categorized as any cataract (including all subtypes of cataract without further sub-classifications) or ARC (being explicitly indicated as the age-related subtype of cataract). Cataract blindness must be bilateral and include operated cases. Since it is generally impossible to obtain the preoperative visual acuity of an operated eye, if one person’s both eyes were operated on for cataract, he/she was presumed to have been bilaterally cataract blind preoperatively; or if only one eye was operated on for cataract and the fellow eye was blind because of cataract at the time of examination, then this person was also presumed to have bilateral cataract blindness [25,34,35]. Those basic assumptions about cataract blindness should be compiled into the included studies that reported the prevalence of cataract blindness. According to different cut-offs of visual acuity for defining blindness, cataract blindness was categorized as cataract blindness by BCVA<0.05, by BCVA<0.10 and by PVA<0.10 respectively.

**Table 1:** The standardized definitions of cataract and cataract blindness in the systematic review

Cataract
1) Lens opacities presenting in at least one eye (unilateral or bilateral);
2) With BCVA≤0.70 in the cataract-affected eye;
3) Including pseudophakia/aphakia.
**Cataract blindness**
1) being blind in the better-seeing eye caused by cataract;
2) Including pseudophakia/aphakia.

#### Study selection and data extraction

All duplicate records within and between different bibliographic databases were identified and eliminated before conducting the formal systematic review. Two independent reviewers (PS and HW) screened titles and abstracts of all retained records. Then the full texts of potentially relevant articles were retrieved and further appraised for inclusion against the eligibility criteria. All discrepancies were resolved through discussions until consensuses were reached.

Finally, relevant data were extracted from the eligible articles by using a pilot tested and refined extraction table, which included three main categories:

1. Characteristics of the study: authors, publication year, study setting, survey year, sampling method, study design (cross-sectional or cohort), case definition and assessment method;
2. Characteristics of the investigated population: sample size, population type (urban or rural), sex (male or female), and age (age range, mean or median age, or midpoint of the age range);
3. Prevalence data: the number of people with cataract/cataract blindness and the number of participants who had been tested, by age group, sex, setting and cataract/cataract blindness subtype where possible.

In cases where stratified prevalence estimates were not available by sex and setting, and only the overall estimates were reported, sex or setting were labelled as “mixed”. Studies were grouped into six geographic regions according to their study sites. Those geographic regions included North China, Northeast China, East China, South Central China, Southwest China and Northwest China, which were delineated by the National Bureau of Statistics of China (**Table 2**) [36-39]. If people in different geographic areas were examined in the same study, prevalence data were extracted for each geographic area separately, where available. For four studies where the years of survey were not specified, three years were subtracted from their publication years to impute the survey years, which was based on the average time lag from survey to publication in studies that provided such information. For studies that reported censoring age groups, eg, older than 80 years, less than 50 years, the missing age band was imputed by taking the same width as other age groups in the same study.

**Table 2:** The six geographic regions in China

Region	Included provinces
North China	Beijing Municipality, Hebei province, Inner Mongolia Autonomous Region, Shanxi province, Tianjin Municipality;
Northeast China	Heilongjiang province, Jilin province, Liaoning province;
East China	Anhui province, Fujian province, Jiangsu province, Jiangxi province, Shandong province, Shanghai Municipality, Zhejiang province;
South Central China	Guangdong province, Guangxi Zhuang Autonomous Region, Hainan province, Henan province, Hubei province, Hunan province;
Southwest China	Chongqing Municipality, Guizhou province, Sichuan province, Tibet Autonomous Region, Yunnan province;
Northwest China	Gansu province, Ningxia Hui Autonomous Region, Qinghai province, Shaanxi province, Xinjiang Uyghur Autonomous Region;

### Statistical analysis

#### Epidemiological modelling of cataract prevalence and pooled prevalence of cataract blindness

Before synthesizing the abstracted data, unadjusted prevalence of cataract (any cataract and ARC) and cataract blindness (by BCVA<0.05, by BCVA<0.10 and by PVA<0.10) was first calculated on the basis of crude numerators and denominators derived from individual studies. In this study, random-effects meta-analysis was performed throughout because sizeable heterogeneity of reported prevalence in different studies was suggested (Table S3 and Figures S1-S5 in **Online Supplementary Document(Online Supplementary Document)**).

For cataract, multiple outcome measurements were available within a single study. To accommodate for this hierarchical data structure (clustering of different stratum-specific prevalence rates from the same study), a multilevel mixed-effects meta-regression model was fitted [40,41]. Before constructing models for estimating the prevalence of cataract, the associations of prevalence rates and study-level variables of interest, ie, age, sex (male and female), setting (urban, rural and mixed), geographic region and survey year were first explored using univariable meta-regression. This was done for any cataract and ARC separately. Age and sex were identified as variables that were commonly associated with the prevalence of both any cataract and ARC (Table S4 in **Online Supplementary Document(Online Supplementary Document)**). For the purpose of producing the national “envelopes” (total number of cases) of any cataract and ARC, the age- and sex-specific prevalence estimates of any cataract and ARC were developed. Given that:

Prevalence = *p* = number of cases/sample size,

Then, the prevalence rates were transformed using a logit link [42]:

logit(*p*) = log_e_(*p*/1 - *p*) = log_e_(*odds*) = α +β_1_ × x_1_ + β_2_ × x_2 _+…β_n_ × x_n_

Given age and sex were the prespecified variables:

logit(*p*) = α + β_1_ × (*age*) + β_2_ × (*sex*)

Thus, the prevalence of any cataract/ARC was:

*P* = e^α+β1 × (^*^age^*^)^ ^+^ ^β2 × (^*^sex^*^)^/1 + e^α + β1 × (^*^age^*^) + β2 × (^*^sex^*^)^

For cataract blindness, the stratum-specific prevalence rates were not universally available in the included studies. Therefore, the pooled prevalence, with 95% confidence intervals (CIs), was obtained from a random-effects meta-analysis model (DerSimonian and Laird method) with inverse-variance weighting. The variance of prevalence was also stabilised with the logit transformation [42]. To check whether a single study disproportionally influenced the pooled results, the sensitivity analysis was conducted by removing one study at a time to run the meta-analysis without it [43]. We also assessed the publication bias by visual inspection of funnel plots test [44].

#### Estimation of the national number of people with cataract and cataract blindness from 1990 to 2015

The total number of people affected by cataract (“cataract envelope”, including post-surgical cases) in China was generated by applying the modelled age- and sex-specific prevalence for each 5-year age group to the corresponding population subgroup in China, available from the United Nations Population Division (UNPD) [45]. This was performed for any cataract and ARC respectively in the years 1990, 2000, 2010 and 2015.

For cataract blindness, the age- and sex-specific prevalence estimates were not specifically constructed, the overall number of people affected by cataract blindness (including post-surgical cases) was, therefore, estimated by multiplying the pooled prevalence of cataract blindness by the national population. This was conducted for the three subgroups of cataract blindness determined by different definitions of blindness (by BCVA<0.05, by BCVA<0.10 and by PVA<0.10) from 1990 to 2015.

#### Projection of the national number of people with cataract and cataract blindness from 2020 to 2050

To derive the projected number of people with cataract/cataract blindness (including post-surgical cases) for the period 2020-2050, the age- and sex-specific prevalence of cataract and the pooled prevalence of cataract blindness were assumed to stay constant over time. For cataract, this assumption was supported by the univariable meta-regression models, where no significant association between survey year and the prevalence of cataract (both any cataract and ARC) was detected (Table S4 in **Online Supplementary Document(Online Supplementary Document)**). The national number of people living with cataract and cataract blindness from 2020 to 2050 was projected by following the same approach as used for deriving the national number of cataract and cataract blindness cases from 1990 to 2015. The demographic projection was taken from the UNPD medium variant population scenario, which was underpinned by assumptions about future fertility and mortality [45,46].

#### Effects of demographic and geographic factors on the prevalence of cataract

Where appropriate (at least seven contributing estimates should be available for each variable [47]), the effects of demographic (ie, age, sex) and geographic features (ie, setting, geographic region) on the prevalence of cataract were initially assessed by univariable meta-regression models. The effect of survey year on the prevalence of cataract was also explored by introducing the survey year into the models. Finally, all variables with p values of less than 0.1 in univariable meta-regression models were entered into the multivariable meta-regression.

#### Estimation of the subnational number of people with cataract from 2000 to 2010

To generate the number of people with cataract in the six geographic regions, an “envelope” approach was adopted. This “envelope” approach was previously endorsed by the Child Health Epidemiology Reference Group (CHERG), and has, since, been widely advocated in the disease burden domain [38,39,48-51]. First, based on the final multivariable meta-regression that took the effects of demographic and geographic features into account, cataract prevalence in each geographic region was calculated respectively. Then the regional cases of cataract were estimated by multiplying the regional cataract prevalence by their corresponding population. Finally, the regional cataract cases were adjusted to fit into the national “cataract envelope”. This was done for the years 2000 and 2010, where the regional population data could be derived from the fifth and sixth censuses respectively [36,37].

All statistical analyses were done with R, version 3.3.0 (R Foundation for Statistical Computing, Vienna, Austria) with the packages meta (version 4.8-4), metaphor (version 2.0-2) and ggplot2 (version 2.2.1) [52]. A two-sided p-value of less than 0.05 was regarded as indicative of statistically significant, unless otherwise specified. All maps were created using ArcMap version 10.1 (Environmental Systems Research Institute, Redlands, CA, USA), based on the China base map (in shapefile format) obtained from the Global Administrative Areas (GADM) database (GADM, 2015, version 2.0; www.gadm.org).

## RESULTS

### Summary of systematic review

**Figure 1:**
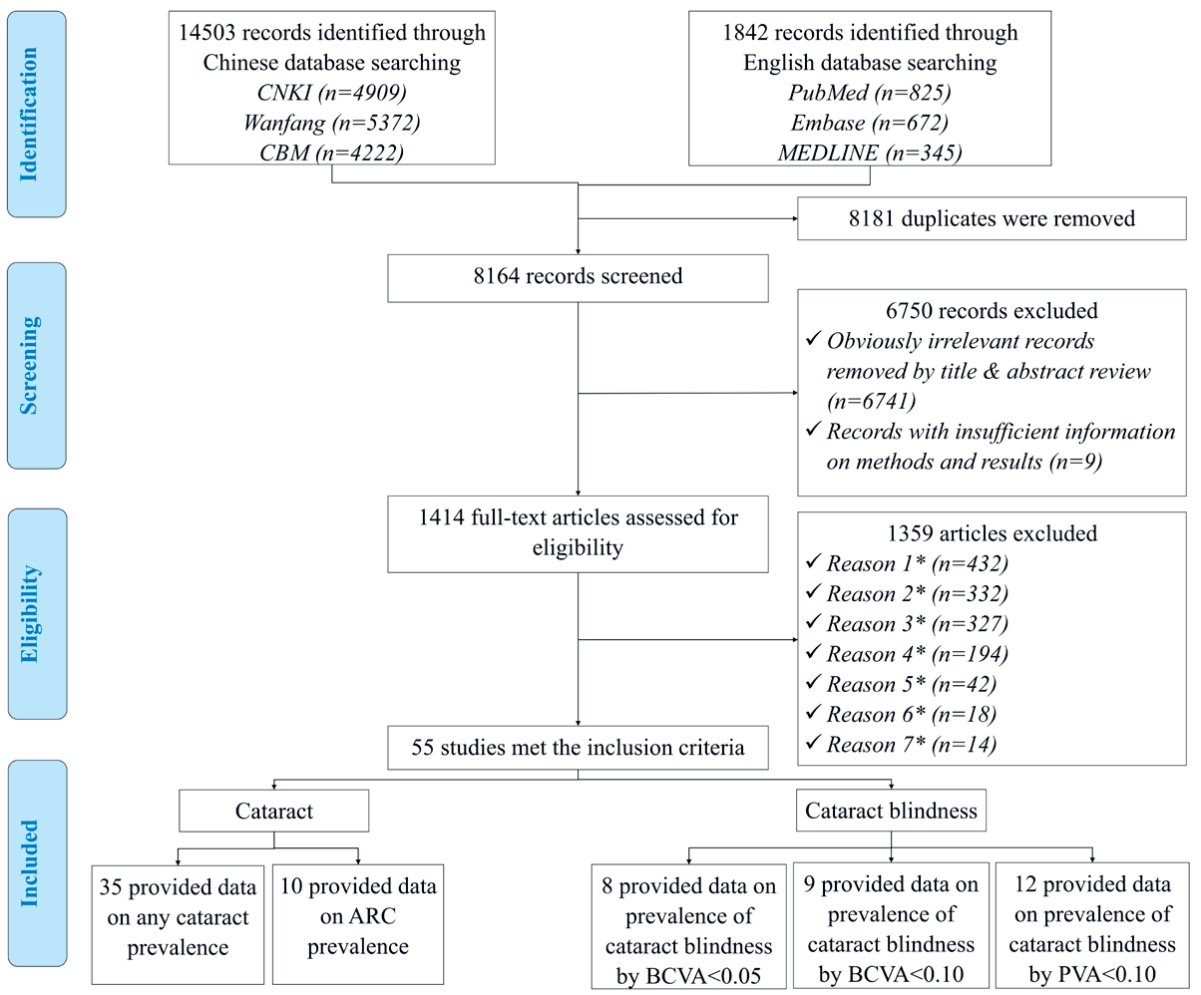
Systematic review flow diagram of studies on cataract and cataract blindness prevalence in China. *Reason 1 – Studies that were conducted in a population with unrepresentative characteristics (hypertensive patients, people with reduced vision, etc.); *Reason 2 – Studies that were not population-based; *Reason 3 – Studies that relied on self-reported diagnoses of cataract or cataract blindness; *Reason 4 – Papers with no numerical prevalence measure of cataract or cataract blindness; *Reason 5 – Studies that didn’t adopt the standardized definitions of cataract and cataract blindness; *Reason 6 – M ultiple publications of the same study; *Reason 7 – Studies that were not based in China.

The initial literature search yielded a total of 16345 citations. After removing 8181 duplicates, the titles and abstracts of 8164 unique records were screened for relevance. Of these, 1414 potentially relevant articles were reviewed in full-text form and 1359 were subsequently excluded. Finally, 55 studies met the eligibility criteria and were included in the systematic review and meta-analysis. The review process is summarized in **Figure 1** as a PRISMA flowchart. A list of the included studies is available in Table S5 in **Online Supplementary Document(Online Supplementary Document)**.

**Figure 2:**
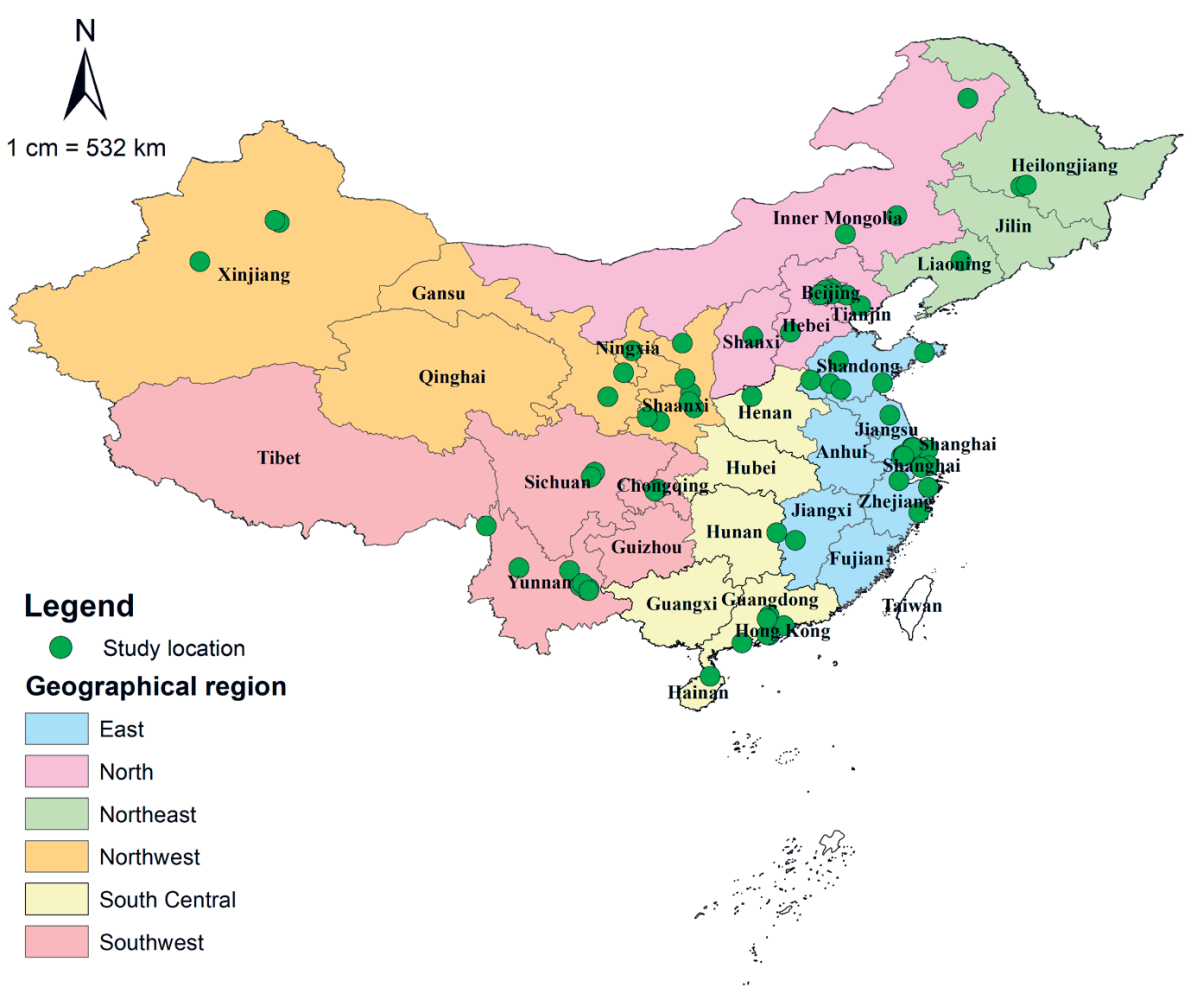
Geographical distribution of the included studies on cataract and cataract blindness prevalence in China (n = 55).

**Table 3** summarises the main characteristics of the 55 included studies. All those studies were cross-sectional in design, among which the majority were published in this decade (from 2010 onwards). Most studies were relatively large, with more than 2000 participants. Almost all studies provided sex-specific data, enabling the exploration of sex difference in cataract prevalence. Of the included studies, 35 studies examined the prevalence of any cataract, ten provided prevalence data on ARC; eight studies focused on the prevalence of cataract blindness by BCVA<0.05, nine on the prevalence of cataract blindness by BCVA<0.10, and 12 on the prevalence of cataract blindness by PVA<0.10. The diverse geographical areas covered by the included studies are shown in **Figure 2**, and the detailed characteristics of all included studies are demonstrated in Table S6 in **Online Supplementary Document(Online Supplementary Document)**.

**Table 3:** Main characteristics of the included studies on cataract and cataract blindness in China (n = 55)

Characteristics	Number of studies provided prevalence data on (%)*				
**Any cataract (n = 35)**	**ARC (n = 10)**	**Cataract blindness by BCVA<0.05 (n = 8)**	**Cataract blindness by BCVA<0.10 (n = 9)**	**Cataract blindness by PVA<0.10 (n = 12)**
**Year published:**					
1990-1999	0 (0.0)	0 (0.0)	0 (0.0)	0 (0.0)	1 (8.3)
2000-2009	10 (28.6)	6 (60.0)	4 (50.0)	0 (0.0)	4 (33.3)
2010-2017	25 (71.4)	4 (40.0)	4 (50.0)	9 (100.0)	7 (58.3)
**Setting:**					
Urban	11 (31.4)	3 (30.0)	3 (37.5)	0 (0.0)	4 (33.3)
Rural	12 (34.3)	4 (40.0)	3 (37.5)	5 (55.6)	5 (41.7)
Mixed	10 (28.6)	2 (20.0)	1 (12.5)	4 (44.4)	2 (16.7)
Both	2 (5.7)	1 (10.0)	1 (12.5)	0 (0.0)	1 (8.3)
**Sex:**					
Mixed	4 (11.4)	0 (0.0)	0 (0.0)	0 (0.0)	0 (0.0)
Both	31 (88.6)	10 (100.0)	8 (100.0)	9 (100.0)	12 (100.0)
**Sample size:**					
≤2000	7 (20.0)	4 (40.0)	1 (12.5)	1 (11.1)	1 (8.3)
2001-5000	11 (31.4)	2 (20.0)	5 (62.5)	1 (11.1)	6 (50.0)
5001–10000	12 (34.3)	3 (30.0)	2 (25.0)	5 (55.6)	4 (33.3)
>10000	5 (14.3)	1 (10.0)	0 (0.0)	2 (22.2)	1 (8.3)
**Geographic region†:**					
North China	6 (17.1)	3 (20.0)	2 (25.0)	2 (15.4)	2 (16.7)
Northeast China	2 (5.7)	1 (6.7)	1 (12.5)	1 (7.7)	0 (0.0)
East China	15 (42.9)	3 (20.0)	2 (25.0)	7 (53.8)	4 (33.3)
South Central China	4 (11.4)	2 (13.3)	0 (0.0)	1 (7.7)	1 (8.3)
Southwest China	3 (8.6)	2 (13.3)	2 (25.0)	1 (7.7)	5 (41.7)
Northwest China	5 (14.3)	4 (26.7)	1 (12.5)	1 (7.7)	0 (0.0)

*Some studies reported the prevalence estimates of any cataract, ARC, cataract blindness by BCVA<0.05, cataract blindness by BCVA<0.10 and cataract blindness by PVA<0.10 simultaneously, therefore the sum of the number of studies exceeded 55.

†Some studies covered more than one geographic region, therefore the sum of coved geographic regions exceeded the sum of studies.

### Age- and sex-specific prevalence of cataract and pooled prevalence of cataract blindness

**Figure 3:**
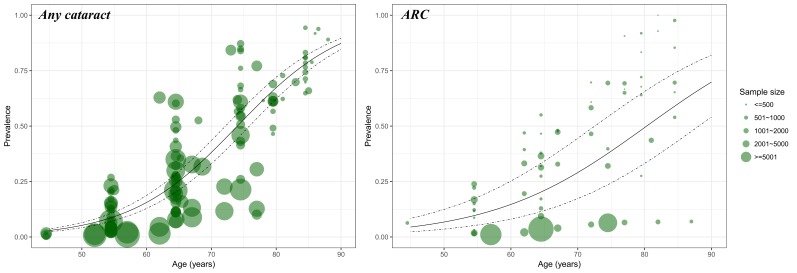
Age-specific prevalence of cataract and ARC based on informative data points from the included studies. Note: The size of each bubble is proportional to the sample size. For cataract, there were 143 data points for constructing the relation between age and prevalence; For ARC, there were 66.

For cataract, a substantial number of data points from the included studies guaranteed our ability to construct the relation between age and prevalence (**Figure 3**). The age range covered by informative data points for any cataract was wider than that for ARC. To ensure the comparability of the estimated prevalence of any cataract and ARC, the age range in this study was set as from 45 to 89 years, where the most informative data points concentrated. The effect of sex on the prevalence of cataract was also assessed based on studies that provided sex-specific cataract prevalence (Table S4 in **Online Supplementary Document(Online Supplementary Document)**).

**Figure 4:**
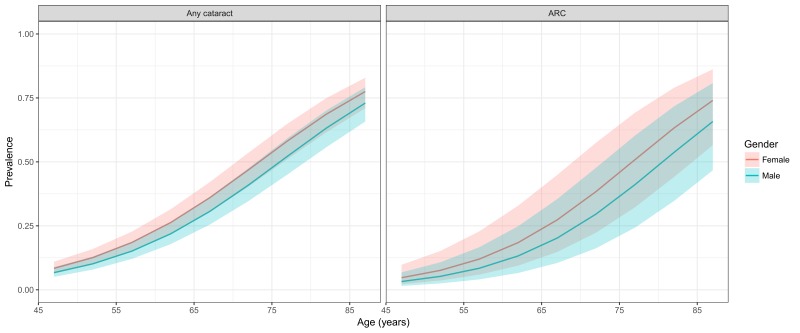
Estimated age- and sex-specific prevalence of any cataract and ARC in China, with 95% confidence intervals.

**Table 4** and **Figure 4** demonstrate the estimated age- and sex-specific prevalence of any cataract and ARC. Generally, the prevalence of any cataract and ARC was higher in females than in males, and both showed a steady rise with advanced age. In males, the prevalence of any cataract ranged from 6.71% (95% CI = 5.06-8.83) in people aged 45-49 years to 73.01% (95% CI = 65.78-79.2) in elderly aged 85-89 years. In females, the prevalence of any cataract increased from 8.39% (95% CI = 6.36-10.98) in individuals aged 45-49 years to 77.51% (95% CI = 71.00-82.90) in those aged 85-89 years. In the case of ARC, the prevalence rates ranged from 3.23% (95% CI = 1.51-6.80) in males aged 45-49 years to 65.78% (95% CI = 46.72-80.82) in those aged 85-89 years. The prevalence of ARC in females was consistently higher than that in males, ranging from 4.72% (95% CI = 2.22-9.76) in the 45-49 years age group to 74.03% (95% CI = 56.53-86.21) in the 85–89 years age group.

**Table 4:** Estimated sex-specific prevalence of any cataract and ARC in China, by age group

Age (years)	Prevalence of any cataract (%, 95% CI)		Prevalence of ARC (%, 95% CI)	
**Male**	**Female**	**Male**	**Female**
45-49	6.71	8.39	3.23	4.72
	(5.06-8.83)	(6.36-10.98)	(1.51-6.80)	(2.22-9.76)
50-54	10.16	12.59	5.25	7.59
	(7.91-12.97)	(9.86-15.95)	(2.48-10.76)	(3.64-15.17)
55-59	15.11	18.49	8.42	12.00
	(12.06-18.77)	(14.88-22.73)	(4.07-16.64)	(5.91-22.84)
60-64	21.89	26.30	13.24	18.46
	(17.86-26.53)	(21.69-31.50)	(6.58-24.87)	(9.46-32.92)
65-69	30.60	35.97	20.22	27.31
	(25.45-36.29)	(30.31-42.05)	(10.47-35.45)	(14.78-44.88)
70-74	40.97	46.92	29.60	38.41
	(34.69-47.55)	(40.36-53.59)	(16.23-47.71)	(22.33-57.50)
75-79	52.20	58.18	41.10	50.86
	(45.07-59.25)	(51.10-64.94)	(24.30-60.28)	(32.25-69.24)
80–84	63.22	68.65	53.67	63.21
	(55.73-70.12)	(61.59-74.93)	(34.67-71.65)	(44.05-78.94)
85-89	73.01	77.51	65.78	74.03
	(65.78-79.20)	(71.00-82.90)	(46.72-80.82)	(56.53-86.21)

**Figure 5:**
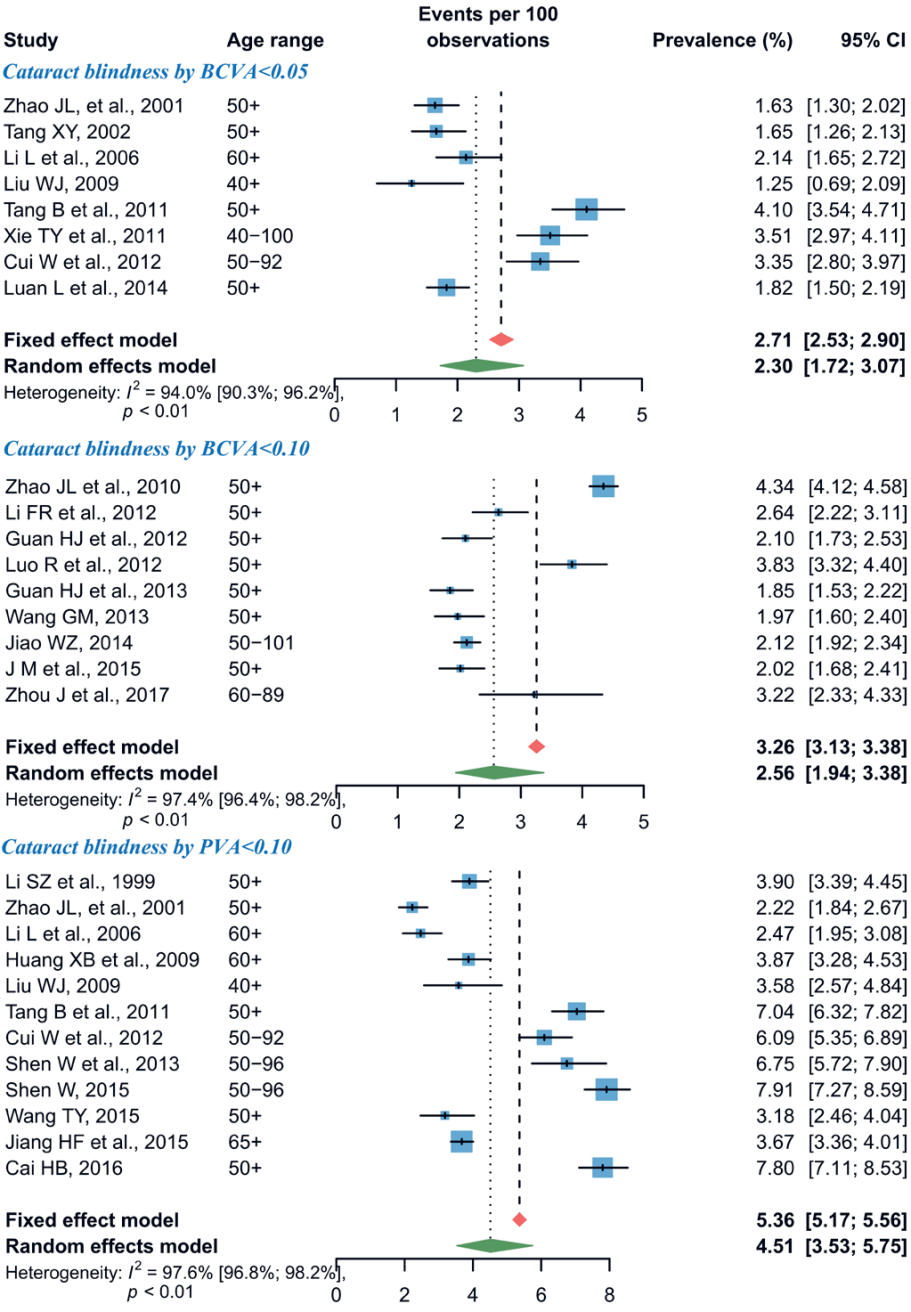
Forest plots of cataract blindness prevalence in China. Note: Data were pooled by a random-effects model; the size of the blue shaded areas is proportional to the weight of each study.

For cataract blindness, the pooled prevalence estimates by random-effects meta-analyses are shown in **Figure 5**. Overall, the pooled prevalence of cataract blindness by BCVA<0.05 was 2.30% (95% CI = 1.72-3.07) and that of cataract blindness by BCVA<0.10 was 2.56% (95% CI = 1.94-3.38). At a more relaxed threshold level for defining blindness, the pooled prevalence of cataract blindness by PVA<0.10 was 4.51% (95% CI = 3.53-5.75). According to the leave-one-out sensitivity analysis, no single study had a substantial influence on the overall prevalence of cataract blindness, where the pooled prevalence of cataract blindness by BCVA<0.05 ranged from 2.11% (95% CI = 1.59-2.81) to 2.45% (95% CI = 1.82-3.30), that of cataract blindness by BCVA<0.10 from 2.38% (95% CI = 1.98-2.87) to 2.67% (95% CI = 2.00-3.55), and that of cataract blindness by PVA<0.10 from 4.28% (95% CI = 3.32-5.49) to 4.81% (95% CI = 3.81-6.06) (Figure S6 in **Online Supplementary Document(Online Supplementary Document)**). The asymmetrical shape of funnel plots revealed some evidence of potential publication bias (Figure S7 in **Online Supplementary Document(Online Supplementary Document)**).

### National number of people with cataract and cataract blindness from 1990 to 2015

**Figure 6:**
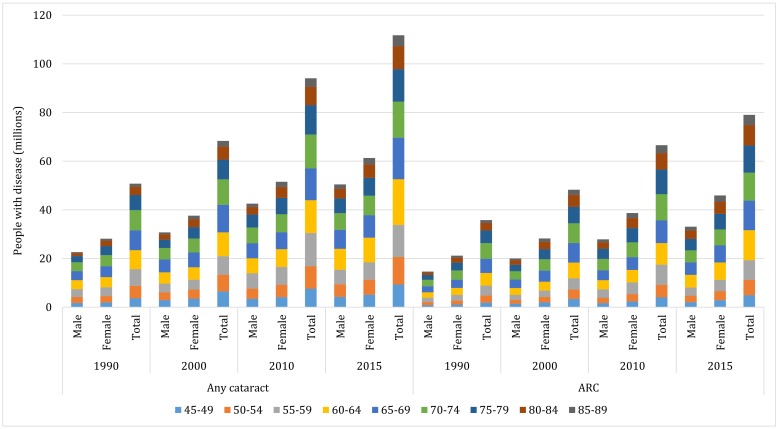
Estimated sex-specific number of people with any cataract and ARC in China from 1990 to 2015, with contributing age groups.

For cataract, the numbers of people with any cataract and ARC were computed by applying the age- and sex-specific prevalence rates to the correspondingly stratified national population aged 45-89 years in the years 1990, 2000, 2010 and 2015 (**Table 5** and Table S7 in **Online Supplementary Document(Online Supplementary Document)**. At the national level, the prevalence of any cataract in people aged 45-89 years slightly decreased by 1.13%, from 22.21% (95% CI = 18.46-26.42) in 1990 to 21.96% (95% CI = 18.26-26.10) in 2015. During this period, the prevalence of ARC in people 45-89 years of age ranged from 15.65% (95% CI = 8.67-26.06) to 15.53% (95% CI = 8.67-25.71), indicating an overall decreasing rate of 0.79%. Despite the gentle decline in prevalence estimates within this time frame, there was a considerable increase in the national number of people with cataract. In people aged 45-89 years, the number of any cataract cases was 50.75 million (95% CI = 42.17-60.37) in 1990 and 111.74 million (95% CI = 92.94-132.84) in 2015, and that of ARC cases rose from 35.77 million (95% CI = 19.81-59.55) in 1990 to 79.04 million (95% CI = 44.14-130.85) in 2015, which yielded overall increasing rates of 120.19% and of 120.96% respectively throughout this period. In 2015, around 71% of the cataract cases were the age-related subtype, and the age group that contributed the most cases was 60-64 years for both any cataract and ARC (**Figure 6**).

**Table 5:** Estimated prevalence and number of people with cataract and cataract blindness in China from 1990 to 2015

Type	Prevalence (%, 95% CI)		Number of people with disease (million, 95% CI)		Relative rate of change (%, 1990-2015)	
**1990**	**2015**	**1990**	**2015**	**Prevalence**	**Cases**
Cataract	22.21	21.96	50.75	111.74	-1.13	+120.19
	(18.46-26.42)	(18.26-26.10)	(42.17-60.37)	(92.94-132.84)		
ARC	15.65	15.53	35.77	79.04	-0.79	+120.96
	(8.67-26.06)	(8.67-25.71)	(19.81-59.55)	(44.14-130.85)		
Cataract blindness (BCVA<0.05)	2.30		5.26	11.71	–	+122.72
	(1.72-3.07)		(3.93-7.02)	(8.75-15.62)		
Cataract blindness (BCVA<0.10)	2.56		5.85	13.03	–	
	(1.94-3.38)		(4.43-7.72)	(9.87-17.20)		
Cataract blindness (PVA<0.10)	4.51		10.31	22.95	–	
	(3.53-5.75)		(8.07-13.14)	(17.97-29.26)		

For cataract blindness, the overall prevalence was assumed to be constant over the period 1990-2015, but even so, an increasing trend in cataract-blind cases was witnessed (**Table 5**). From 1990 to 2015, the number of people with cataract blindness by BCVA<0.05 (aged 45-89 years) increased from 5.26 million (95% CI = 3.93-7.02) to 11.71 million (95% CI = 8.75-15.62), and those with cataract by BCVA<0.10 rose from 5.85 million (95% CI = 4.43-7.72) to 13.03 million (95% CI = 9.87-17.20). By using the cut-off of PVA<0.10 for defining blindness, the number of people with cataract blindness (aged 45-89 years) ranged from 10.31 million (95% CI = 8.07-13.14) to 22.95 million (95% CI = 17.97-29.26) within the same time frame.

### Projection of national number of people with cataract and cataract blindness from 2020 to 2050

**Figure 7:**
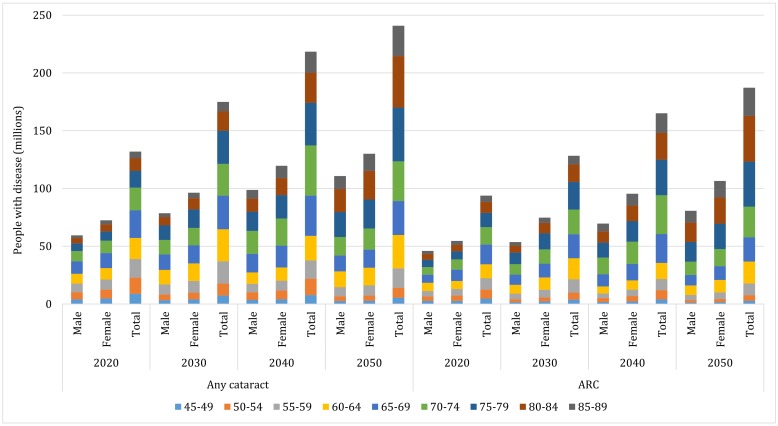
Projected sex-specific number of people with any cataract and ARC in China from 2020 to 2050, with contributing age groups.

For cataract, the projection of national affected cases from 2020 to 2050 was based on the assumption that the age- and sex-specific prevalence of any cataract and ARC would remain constant over time. By extrapolating the estimated age- and sex-specific prevalence of any cataract and ARC to the UNPD data, the numbers of people with any cataract and ARC were projected up to the year 2050 (**Table 6** and Table S7 in **Online Supplementary Document(Online Supplementary Document)**). Given the demographic changes over the next three decades, the prevalence of any cataract in people aged 45-89 years is expected to increase from 22.78% (95% CI = 18.98-27.03) to 33.34% (95% CI = 28.53-38.40), and that of ARC from 16.21% (95% CI = 9.07-26.72) to 25.93% (95% CI = 15.67-38.94) between the years 2020 and 2050, which will indicate increasing rates of 46% and 60% respectively. In people aged 45-89 years, the total number of affected cases is projected to keep increasing, with the number of those with ARC at a higher rate than those with any cataract (100% vs 83%). In 2020, the total number of people with any cataract (aged 45-89 years) will be 131.92 million (95% CI = 109.91-156.49), and this figure is projected to increase to 240.83 million (95% CI = 206.07-277.35) by the year 2050. Among them, the number of people with ARC is projected to grow relatively rapidly from 93.83 million (95% CI = 52.52-154.69) in 2020 to 187.26 million (95% CI = 113.17-281.23) in 2050. By then, ARC will account for 78% of all cataract cases in China, and the age group where most cases concentrate will be 75-79 years for any cataract and 80-84 years for ARC (**Figure 7**).

**Table 6:** Projected prevalence and number of people with cataract and cataract blindness in China from 2020 to 2050

Type	Prevalence (%, 95% CI)		Number of people with disease (million, 95% CI)		Relative rate of change (%, 2020-2050)	
**2020**	**2050**	**2020**	**2050**	**Prevalence**	**Cases**
Cataract	22.78	33.34	131.92	240.83	+46.35	+82.56
	(18.98-27.03)	(28.53-38.40)	(109.91-156.49)	(206.07-277.35)		
ARC	16.21	25.93	93.83	187.26	+59.98	+99.57
	(9.07-26.72)	(15.67-38.94)	(52.52-154.69)	(113.17-281.23)		
Cataract blindness (BCVA<0.05)	2.30		13.32	16.61	–	+24.75
	(1.72-3.07)		(9.96-17.78)	(12.42-22.17)		
Cataract blindness (BCVA<0.10)	2.56		14.82	18.49	–	
	(1.94-3.38)		(11.23-19.57)	(14.01-24.41)		
Cataract blindness (PVA<0.10)	4.51		26.11	32.58	–	
	(3.53-5.75)		(20.44-33.29)	(25.50-41.53)		

For cataract blindness (**Table 6**), the number of people with cataract blindness by BCVA<0.05 (aged 45-89 years) is projected to rise from 13.32 million (95% CI = 9.96-17.78) in 2020 to 16.61 million (95% CI = 12.42-22.17) in 2050, while those with cataract by BCVA<0.10 (aged 45-89 years) from 14.82 million (95% CI = 11.23-19.57) to 18.49 million (95% CI = 14.01-24.41) during this period. When applying a less stringent cut-off of PVA<0.10 for blindness, the number of people with cataract blindness (aged 45-89 years) is expected to grow from 26.11 million (95% CI = 20.44-33.29) in 2020 to 32.58 million (95% CI = 25.50-41.53) in 2050.

### Effects of demographic and geographic factors on the prevalence of cataract

Results from the univariable meta-regression analyses revealed that age and sex were significantly associated with the prevalence of both any cataract and ARC, with older people and females having higher prevalence rates. No secular trend or urban-rural difference in the prevalence of any cataract or ARC were revealed (Table S4 in **Online Supplementary Document(Online Supplementary Document)**). In addition, a weaker association was seen for the geographic distribution of any cataract prevalence, where people living in North China were suggested to be with a relatively lower prevalence than those in East China (*P* < 0.1). For the purpose of constructing a multivariable regression model, insufficient data were available for ARC. Therefore, a multivariable regression model that took the effects of age, sex and geographic region simultaneously was only developed for any cataract:

logit(*p*) = –6.740 + 0.091 × *Age*+(–0.242) × *Gender_male_*+*U_region_*

Where indicates the prevalence of any cataract; refers to the absolute value of age,  = 1 for males and = 0 for females; represents the region-level effect, which equals to 0 for East China, -0.269 for North China, 0.486 for Northeast China, -0.075 for Northwest China, 0.636 for South Central China and 0.125 for Southwest China.

### Regional number of people with any cataract from 2000 to 2010

Based on the final formula for developing the age- and sex-specific prevalence of any cataract in different regions, the national cases of any cataract in the years 2000 and 2010 were respectively allocated into the six geographic regions in China (**Table 7**). In 2000, the prevalence of any cataract in people aged 45-89 years was 21.62% (95% CI = 17.97-25.72) in China, varying from 15.05% (95% CI = 11.39-19.43) in North China to 28.38% (95% CI = 23.80-32.92) in South Central China. In 2010, the national prevalence of any cataract in people aged 45-89 years rose to 21.96% (95% CI = 18.26-26.11). The region with the highest prevalence of any cataract was still South Central China (28.50% [95% CI = 23.91-33.07]) and that with the lowest prevalence of any cataract was still North China (15.03% [95% CI = 11.39-19.43]). During 2000-2010, the prevalence of any cataract slightly increased by 1.57%, with the most pronounced increase occurring in Southwest China (7.31%). Northeast China was the only region with a declining rate in the prevalence of any cataract (0.11%) during this period.

**Table 7:** Estimated prevalence and number of people with any cataract in China from 2000 to 2010, by geographic region

Region	Prevalence of any cataract (%, 95% CI)		Number of people with any cataract (million, 95% CI)		Relative rate of change (%, 2000-2010)	
**2000**	**2010**	**2000**	**2010**	**Prevalence**	**Cases**
North China	15.05	15.03	5.62	8.13	-0.11	+44.53
	(11.39-19.43)	(11.39-19.43)	(4.26-7.26)	(6.16-10.50)		
Northeast China	24.14	24.57	6.80	10.30	+1.77	+51.34
	(15.86-33.25)	(16.20-33.80)	(4.47-9.37)	(6.79-14.16)		
East China	19.36	19.53	18.89	25.72	+0.88	+36.17
	(18.85-20.32)	(19.05-20.49)	(18.39-19.83)	(25.09-26.98)		
South Central China	28.38	28.50	23.50	31.79	+0.41	+35.25
	(23.80-32.92)	(23.91-33.07)	(19.70-27.26)	(26.67-36.88)		
Southwest China	20.24	21.72	10.14	13.28	+7.31	+30.94
	(14.75-26.47)	(16.01-28.02)	(7.39-13.26)	(9.79-17.13)		
Northwest China	16.83	17.33	3.37	4.87	+3.00	+44.30
	(12.88-21.51)	(13.34-22.01)	(2.58-4.31)	(3.75-6.18)		
China	21.62	21.96	68.33	94.07	+1.57	+37.67
	(17.97-25.72)	(18.26-26.11)	(56.79-81.29)	(78.24-111.84)		

**Figure 8:**
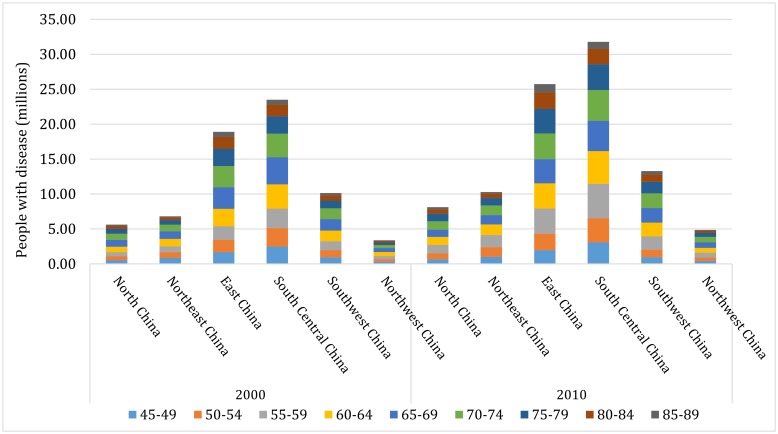
Estimated regional number of people with any cataract and contributing age groups in the years 2000 and 2010.

As demonstrated in **Table 7** and **Figure 8**, a total of 68.33 million (95% CI = 56.79-81.29) people aged 45-89 years were affected by any cataract in 2000. Among them, more than one-third (34%) were living in South Central China (23.50 million, 95% CI = 19.70-27.26) and only 5% were in Northwest China (3.37 million, 95% CI = 2.58-4.31). With the demographic ageing trend in China, the national number of people (aged 45-89 years) with any cataract reached to 94.07 million (95% CI = 78.24-111.84) by 2010. At the regional level, the number of people with any cataract was still the largest in South Central China (31.79 million, 95% CI = 26.67-36.88) and the smallest in Northwest China (4.87 million, 95% CI = 3.75-6.18)-equivalent to 34% and 5% of the national cases of any cataract in 2010 respectively. Over the decade from 2000 to 2010, the number of people with any cataract increased by 38% at the national level. For the six geographic regions, the most marked increase was seen in Northeast China (51%) and the least in Southwest China (31%).

## DISCUSSION

By using all published evidence on the prevalence of cataract and cataract blindness in general Chinese population, this systematic review and meta-analysis, for the first time, provides the most comprehensive figures for the prevalence of cataract and cataract blindness in China at both the national and subnational levels. The data-driven estimates show that the number of people affected by cataract and cataract blindness in China is dramatic. From 1990 to 2015, more than one in five (around 22%) people aged 45-89 years were affected by any cataract, representing a total of 50.75 million and 111.74 million cases in the years 1990 and 2015 respectively. Among them, around 71% were with the age-related subtype of cataract. For cataract blindness, the pooled prevalence varied substantially according to different cutoffs for defining blindness, from 2.30% for cataract blindness by BCVA<0.05 to 4.51% for cataract blindness by PVA<0.10. As the Chinese population ages, the prevalence of cataract and the number of affected people will correspondingly increase in years to come. By the year 2050, the national cases of any cataract in people aged 45-89 years are projected to more than double to 240.83 million, giving a prevalence of one-third (33.34%). At that time, ARC will account for 78% of all cataract cases in China. Moreover, substantial geographic variations were also highlighted in the prevalence of any cataract, which was the highest in South Central China and the lowest in North China during 2000-2010. Meanwhile, in terms of the number of people affected by any cataract, the largest share of cases was in South Central China, while the smallest in Northwest China.

This systematic review and meta-analysis provides the first and most complete description of the prevalence of cataract and cataract blindness in China, which can be used as the basis for the formulation of public-health strategies to ease the burden of cataract and cataract blindness. The strengths of this study include comprehensive and reproducible search strategies incorporated both Chinese and English databases, a dual review process and rigorous selection criteria, which reduced the potential for information bias due to selection and methodological heterogeneity to a minimum. During the systematic review process, only studies that were conducted in general Chinese population were included, thus the generalizability of the whole results presented in this study was largely ensured. With an aim of enhancing the comparability of prevalence estimates from different individual studies, standardized definitions of cataract and cataract blindness based on the available evidence were specified, which made the quantitative synthesis of prevalence rates possible. Moreover, the included studies that reported the prevalence of any cataract were sufficient and with a large geographical diversity, guaranteeing our ability to synthesize the prevalence of any cataract at the subnational level in China.

However, our study is still subject to several potential limitations. First, despite our extensive efforts on minimizing variations by means of strict eligibility criteria and unified definitions, meta-analyses of observational studies are generally vulnerable to bias and confounders inherent in the component studies [53]. Second, in this study, the prevalence of cataract was estimated based on multilevel mixed-effects meta-regression models, by taking the effects of both demographic and geographic features into account. However, the examined demographic and geographic features were cluster-level data, any differences in effects at the individual level might be hidden. Other potential risk factors for cataract, such as economic status, smoking and diabetes, could not be included in the models owing to the absence of relevant information. As a further limitation, the prevalence of cataract could only be estimated at the regional level at best, adequate estimates at the provincial level were not attainable because insufficient studies were from each province in China. For cataract blindness, the roughly pooled estimates of prevalence were based on scant epidemiological evidence, and the limited availability of data points restricted further exploration of heterogeneity and bias by sub-group meta-analysis or meta-regression, therefore several key differences pertinent to variations in risk factors, such as age and sex, might have been obscured. In terms of the cataract blindness prevalence, the assumption that all cataract-operated eyes were blind preoperatively might lead to an overestimation. Third, although the standardized definitions of cataract and cataract blindness served as a basis for our meta-analysis, on the other hand, cataract was not defined primarily based on any specific grading systems of cataract, but rather according to lens opacity in conjunction with reduced visual acuity. This approach, however, does not accord with modern grading systems, such as the Lens Opacities Classification System (LOCS), the Wisconsin Cataract Grading System (WCGS) and the Age-Related Eye Disease Study (AREDS) system, consequently the comparison of the estimates in this study with studies in other western countries would be difficult [54-60]. Of particular note, the cataract/cataract blindness cases referred to people who were suffering from cataract/cataract blindness and those who had already been operated. The currently prevalent cases of cataract/cataract blindness were not able to be estimated with our research approach. Therefore, the national and subnational prevalence of cataract and cataract blindness presented in this study could only be compared with studies (both domestic and international) that adopted similar definitions of cataract and cataract blindness, hindering the generalization of our results to some extent. Fourth, in the projection analysis, the prevalence of cataract and cataract blindness was assumed to remain constant over time. In fact, any changes of risk exposure in the future might increase or decrease the incidence of cataract, and implementation of preventive care might substantially delay the onset of cataract or cataract blindness. The rise of prevalence and number of affected people as indicated in this study mainly relied on the UNPD demographic statistics, and therefore could be argued to be a reflection of population ageing. With the above limitations in mind, several findings in this study should be interpreted with considerable caution.

In a synthesized analysis for estimating the prevalence of cataract in the United States (US), it was estimated that 20.5 million (17.2%) Americans older than 40 years had cataract in either eye in 2000, including those who had pseudophakia/aphakia [55]. In this study, a higher cataract prevalence of 22% was observed in Chinese people aged 45-89 years, corresponding to 68 million people living with cataract in the year 2000. The higher prevalence of cataract in China compared with that in Americans seems to stand by previous statements that cataract is more prevalent in Asians than in Western populations [3,61,62]. When compared with studies using comparable measurements of cataract blindness, the prevalence of cataract blindness by PVA<0.10 was noted to be slightly higher in China (4.5%) than in Nepal (4.1%) [34]. Given the large population size in China, this prevalence rate of cataract blindness actually represents a large group of people who need surgery (if they have not received) and extensive care from both household and community [4,63].

As revealed in our study and many previous studies, the prevalence of any cataract and ARC grows dramatically with advanced age and reaches the highest in older people. The prominent role of ageing as a cause of cataract has also been reinforced in this study, with increased age being the constant risk factor for both any cataract and ARC [1,3,61,64-66]. After age-adjustment, females were found to exhibit a greater tendency to develop cataract and ARC than males, which accords with a large body of evidence across racial groups [3,61,65,66]. Although the mechanism behind sex disparity in cataract formation has not been fully clarified, the protective effect of hormone therapy in cataract development as suggested in previous studies offers a clue for future research [1,67,68]. Taken the effects of ageing population and women’s greater longevity together, the numbers of people living with any cataract and ARC in China will both more than double from 2015 to 2050 based on our projection analysis. Cataract, therefore, will continue to be a leading public health concern and pose wide-ranging effects on its social, economic and health systems.

In view of the geographic patterns of cataract in China, the variations in the prevalence of cataract in different geographic regions could be speculated as a combined result of different distributions of risk factors across the whole country. It has long been suggested that cataractogenesis is a multifactorial process, where individual factors, environmental factors and genetic factors all play a role [1,3]. Risk factors with robust evidence on cataract mainly include advanced age, female sex, smoking, excessive UV B (UVB) exposure, diabetes, etc. [1,3,69-71]. As revealed by our analysis, the geographic region with the highest prevalence of cataract was South Central China in the years of 2000 and 2010. A possible reason is the relatively lower latitude (as a surrogate for a higher UVB exposure level) in south China, but it is still not absolutely clear whether any other factors have contributed to this because relevant information was not universally examined in included studies.

With rapidly ageing populations in China, the question of how best to care for older people with functional impairment is an ever-present concern [4,29,63]. The large cases of cataract and cataract blindness, and the uneven distribution across the nation, suggest that efforts to ameliorate the current and future burden of cataract are urgently needed. In China, the cataract surgical rate (cataract operations per million population per year) has been reported to be 446 in 2004, which was almost the lowest among Asian countries [63,72,73]. This might be resulted by the shortage of qualified eye care practitioners and health resources, in the meanwhile, the uncertain prognosis might also hider affected people to approach such operations [63,74,75]. The estimates presented in this study should be interpreted with the most recent cataract surgical rate in China (if available) to better inform policymakers and health care providers for adequate preparation for the increasing burden of cataract.

Our data also have important implications for adding new evidence into research. In the past decades, despite the advance in diagnostic and surgical techniques, uncertainties and discrepancy in the definitions and assessments of cataract still largely remain in epidemiological studies [1,3]. Until improved large-scale data using comparable definitions and assessments of cataract become available, the systematic review and meta-analysis approach will still be an important option for providing inputs by synthesizing available evidence. This is also the case in China, where improvement in the quality of population-level information on the prevalence of cataract should be a priority for the national statistical agency, notably detailed age categories, classification of different subtypes of cataract, international grading systems, should be adopted as are often the cases. With the emergence of high-quality epidemiological studies on cataract in the foreseen future, reliable prevalence estimates of cataract, and its subtypes could be well achieved. Further, although no confirmed methods to prevent cataract formation are being advocated, well-established risk and protective factors demonstrate possible pathways for slowing cataract progression, such as smoking cessation, wearing hats and sunglasses [1,3]. Given the impact of cataract, the need for further research on preventing and delaying disease is still highlighted.

## CONCLUSIONS

In conclusion, the first and most comprehensive estimates of the prevalence of cataract and cataract blindness in China were provided in this study, at both the national and subnational levels. The prevalence of cataract varied considerably among different demographic and geographic groups. In the coming decades, cataract and cataract blindness will continue to be a leading public-health issue in China. Future work should be prioritized on the promotion of high-quality epidemiological studies on cataract.
