# Supplementary material for: The national and subnational prevalence of cataract and cataract blindness in China: a systematic review and meta-analysis
Source: J Glob Health. 2018 Jun 16;8(1):010804. doi: 10.7189/jogh.08-010804 (PMC6005639; doi:10.7189/jogh.08-010804)
Supplement: Online Supplementary Document [file jogh-08-010804-s001.pdf]

Online Supplementary Document

Song et al. The national and subnational prevalence of cataract and cataract blindness in China

J Glob Health 2018;8:010804

Table S1. Search strategy to identify studies reporting the prevalence of cataract and cataract blindness in China

CNKI

Access Date: 21 Nov 2017

Subject category: Medicine & Public Health

Sub-database: Journal, Featured journal, Doctoral dissertation, Master dissertation

检索表达式：

(SU % '白内障') AND (SU % '发病率' + '发生率' + '患病率'+ '罹患率' + '现患率'+ '死亡率' + '病死率'+ '流行' + '负担'+ '现况调查'+ '现况研究')

发表时间：从 1990-01-01 到 2017-11-21

Search Terms: (SU % 'baineizhang') AND (SU % 'fabinglv' + 'fashenglv' + 'huanbinglv'+ 'lihuanlv' + 'xianhuanlv'+ 'siwanglv' + 'bingsilv'+ 'liuxing' + 'fudan'+ 'xiankuangdiaocha'+ 'xiankuangyanjiu')

Published time：From 01/01/1990 to 21/11/2017

Wanfang

Access Date: 21 Nov 2017

Sub-database: Journal articles, Dissertations

检索表达式：(主题:(白内障)) * (主题:(发病率) + 主题:(发生率) + 主题:(患病率) +主题:(罹患率) + 主题:(现患率) + 主题:(死亡率) +主题:(病死率) + 主题:(流行) + 主题:(负担)+ 主题:(现况调查) + 主题:(现况研究))

时间：1990-2017

Search Terms: (subject: (baineizhang))* (subject: (fabinglv) + subject: (fashenglv) + subject: (huanbinglv)+ subject: (lihuanlv) + subject: (xianhuanlv) + subject: (siwanglv)+ subject: (bingsilv) + subject: (liuxing) + subject: (fudan)+ subject: (xiankuangdiaocha) + subject: (xiankuangyanjiu))

Date: 1990-2017

CBM-SinoMed

Access Date: 21 Nov 2017

Journal category: All journals

检索表达式：

(白内障) AND (发病率 or 发生率 or 患病率 or 罹患率 or 现患率 or 死亡率 or 病死率 or 流行 or 负担 or 现况调查 or 现况研究)

时间：1990-2017

Search Terms: ((baineizhang))* ((fabinglv) OR (fashenglv) OR (huanbinglv) OR (lihuanlv) OR (xianhuanlv) OR (siwanglv) OR (bingsilv) OR (liuxing) OR (fudan) OR (xiankuangdiaocha) OR (xiankuangyanjiu))

Date: 1990-2017

PubMed

Access Date: 22 Nov 2017

Search Terms:

((cataract*) AND (China OR Chinese OR Hongkong OR Macau OR Taiwan) AND (inciden* OR prevalen* OR morbidity OR mortality OR epidemiology)) AND ("1990/01/01"[Date - Publication] : "2017/11/22"[Date - Publication])

Embase (Ovid)

Access Date: 22 Nov 2017

#	Searches
**1**	cataract*.mp. or exp cataract/
**2**	China.mp. or exp China/
**3**	exp Chinese/ or Chinese.mp.
**4**	Hong Kong.mp. or exp Hong Kong/
**5**	Macau.mp. or exp Macau/
**6**	Taiwan.mp. or exp Taiwan/
**7**	exp incidence/ or inciden*.mp.
**8**	exp prevalence/ or prevalen*.mp.
**9**	morbidity.mp. or exp morbidity/
**10**	exp mortality/ or Mortality.mp.
**11**	exp epidemiology/ or Epidemiology.mp.
**12**	2 or 3 or 4 or 5 or 6

**13**	7 or 8 or 9 or 10 or 11
**14**	1 and 12 and 13
**15**	limit 14 to yr="1990 -Current"

Medline (Ovid)

Access Date: 22 Nov 2017

Search Terms:

#	Searches
**1**	exp Cataract/ or cataract*.mp.
**2**	China.mp. or exp China/
**3**	Chinese.mp.
**4**	Hong Kong.mp. or exp Hong Kong/
**5**	Macau.mp. or exp Macau/
**6**	Taiwan.mp. or exp Taiwan/
**7**	exp Incidence/ or inciden*.mp.
**8**	exp Prevalence/ or prevalen*.mp.
**9**	Morbidity.mp. or exp Morbidity/
**10**	Mortality.mp. or exp Mortality/
**11**	Epidemiology.mp. or exp Epidemiology/
**12**	2 or 3 or 4 or 5 or 6
**13**	7 or 8 or 9 or 10 or 11
**14**	1 and 12 and 13
**15**	limit 14 to yr="1990 -Current"

Table S2. A "data microarray" illustrating the definitions of cataract and cataract blindness adopted in population-based studies in China

Study ID	Study	Cataract type		Blindness				Cataract definition						
		Any cataract	ARC	PVA< 0.10	PVA< 0.05	BCVA< 0.10	BCVA< 0.05	Lens opacities	PVA< 0.70	BCVA≤ 0.70	BCVA< 0.63	BCVA< 0.60	Including operated cases	LOCS grading
CA-01	Li S et al., 1999													
CA-02	Zhao JL, et al., 2001													
CA-03	Tang X, 2008													
CA-04	Tang XY, 2002													
CA-05	Wang YX et al., 2003													
CA-06	Li ZQ et al., 2004													
CA-07	Chen L et al., 2004													
CA-08	Ding L et al., 2005													
CA-10	Li ZJ et al., 2007													
CA-12	Zhou Y et al., 2011													
CA-14	Xie TY et al., 2011													
CA-15	Zhao MG et al., 2011													
CA-16	Zhang HF, 2013													
CA-17	Li FR et al., 2012													
CA-18	Bi JX, 2012													
CA-19	Guan HJ et al., 2012													
CA-20	Luo R et al., 2012													
CA-21A	Guan HJ et al., 2013													
CA-21B	J M et al., 2015													
CA-22	Cai N et al., 2013													
CA-23A	Jiao WZ, 2014													
CA-23B	Wang GM, 2013													
CA-23C	Tang HY, 2012													
CA-24	Tian F et al., 2014													

CA-25A	Shen W, 2015													
CA-25B	Shen W et al., 2013													
CA-26	Wang TY, 2015													
CA-27	Jiang HF et al., 2015													
CA-28	Long XX, 2015													
CA-29	Hu Y et al., 2016													
CA-32	Yao HY et al., 2012													
CA-33	Gao RF et al., 2012													
CA-34	Geng JQ, 2017													
CA-35	Luan L et al., 2014													
CA-36	Zhao J, et al., 2010													
CA-37	Fu YZ, et al., 2005													
CA-38	Xu P, 2003													
CA-39	Wu SF, et al., 2015													
CA-30	Cai HB, 2016													
CA-09	Li L, et al., 2006													
CA-11	Huang XB, et al., 2009													
CA-13	Tang B, et al., 2011													
CA-31	Zhou J, et al., 2017													
ARC-9	Liu WJ, 2009													
ARC-6	Cui W, et al., 2012													
ARC-1A	Peng YS et al., 2007													
ARC-1B	Quan YL et al., 2006													
ARC-2	Feng J et al., 2008													
ARC-3	Bai JS et al., 2012													
ARC-4	Xiang W et al., 2015													
ARC-5	Zheng H et al., 2001													
ARC-7	Zeng H et al., 2011													

ARC-8	Sheng Y et al., 2016													
ARC-10	Chang L et al., 2009													
ARC-11	Yong Y, 2001													

Note: The full reference list can be found in Table S5.

Table S3. Meta-analysis of the prevalence of cataract and cataract blindness for assessing heterogeneity between studies

Heterogeneity across included studies was assessed using the $\chi 2$ test on Cochran's Q statistic, and quantified using the I^2 statistics, with a two-sided p value of less than 0.05 being indicative of heterogeneity between studies in Q statistic and I^2 represents the proportion of total variation that is due to heterogeneity rather than chance (with values of <25%, 25–75%, and >75% indicating low, moderate and high heterogeneity, respectively) [1, 2]. As shown in Figures S1-S5, significant high heterogeneity was detected between studies that reported prevalence rates of any cataract (I^2=99.8% [95% CI: 99.8-99.9], p<0.0001), ARC (I^2=99.9% [95% CI: 99.9-99.9], p<0.0001), cataract blindness by BCVA<0.05 (I^2=94.0% [95% CI: 90.3-96.2], p<0.0001), cataract blindness by BCVA<0.10 (I^2=97.4% [95% CI: 96.4-98.2], p<0.0001) and cataract blindness by PVA<0.10 (I^2=97.6% [95% CI: 96.8-98.2], p<0.0001).

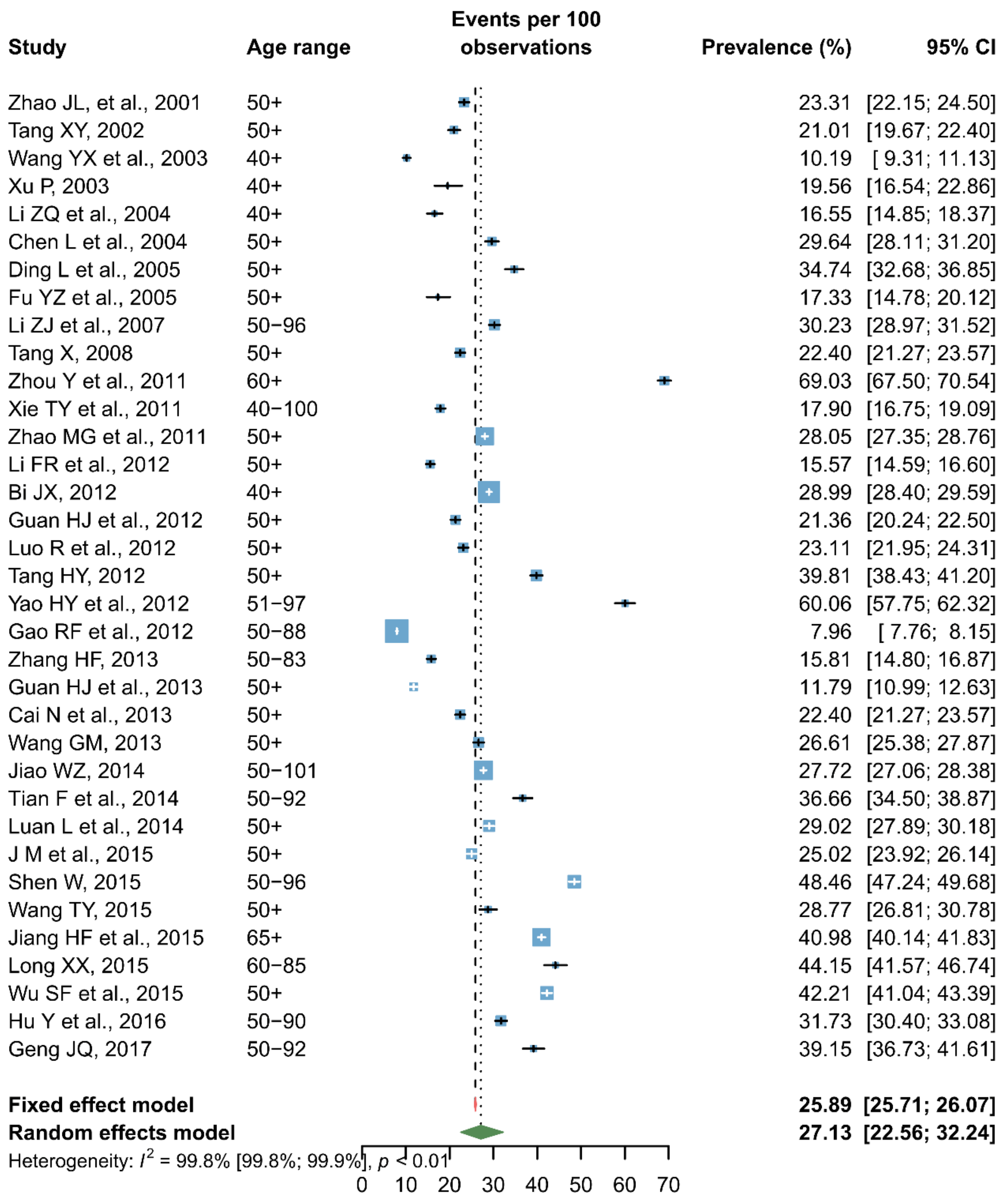

Figure S1. Meta-analysis of the prevalence of any cataract (n=35)

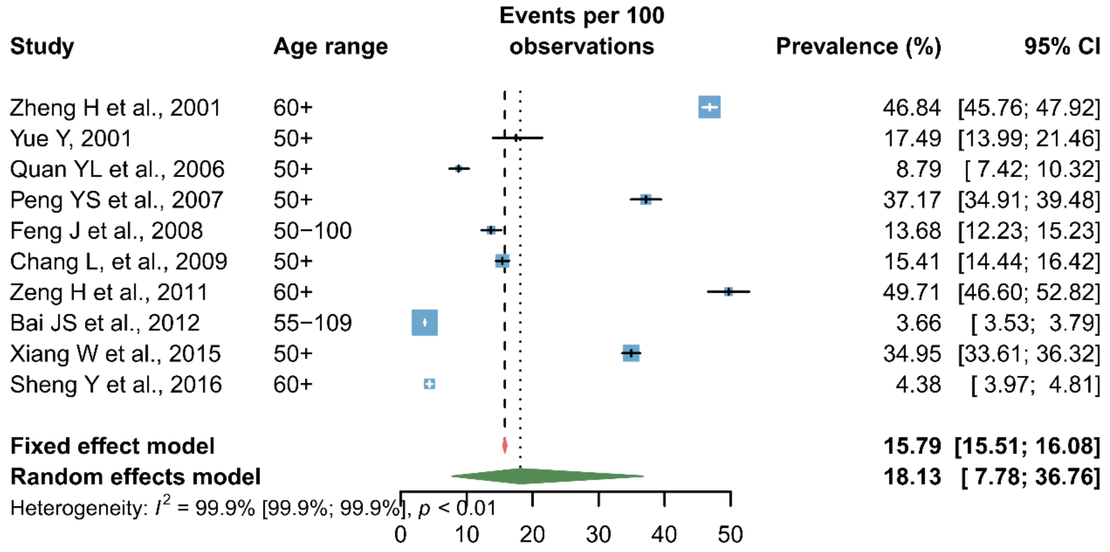

Figure S2. Meta-analysis of the prevalence of ARC (n=10)

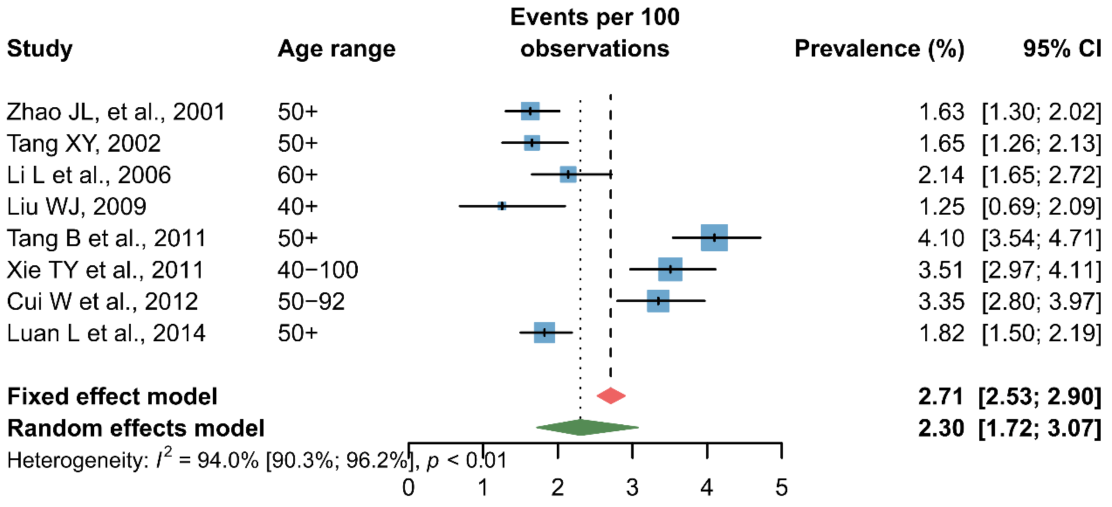

Figure S3. Meta-analysis of the prevalence of cataract blindness by BCVA<0.05 (n=8)

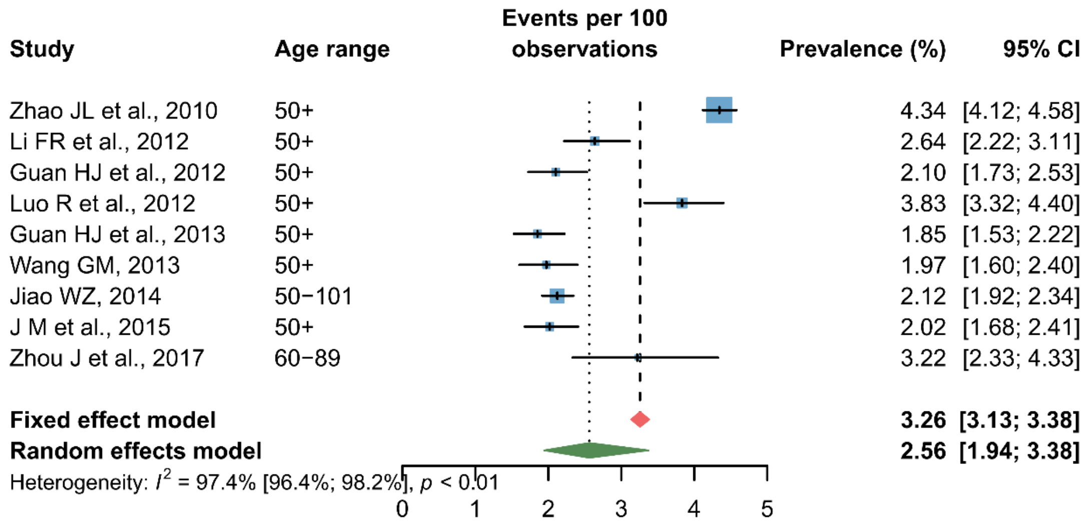

Figure S4. Meta-analysis of the prevalence of cataract blindness by BCVA<0.10 (n=9)

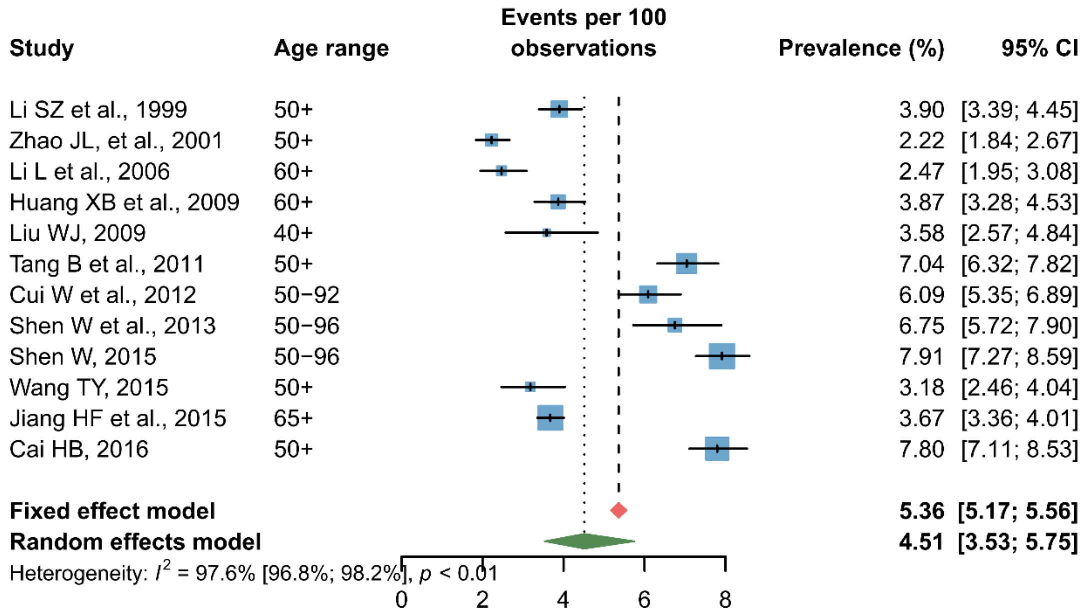

Figure S5. Meta-analysis of the prevalence of cataract blindness by PVA<0.10 (n=12)

Table S4. Unadjusted odds ratios for any cataract and ARC in terms of demographic and geographic factors from univariable meta-regression models, with 95% confidence intervals

Variable	Any cataract		ARC	
	Number of studies	OR (95% CI)	Number of studies	OR (95% CI)
Age (per decade increase)	35	3.34 (3.29-3.40)*	10	2.74 (2.65-2.84)*
Sex$				
Female	29	Reference	10	Reference
Male	29	0.79 (0.77-0.80)*	10	0.67 (0.64-0.71)*
Setting				
Mixed	10	Reference	2	Reference
Rural	14	1.35 (0.86-2.12)	5	1.19 (0.20-7.12)
Urban	13	0.76 (0.49-1.20)	4	1.71 (0.25-11.61)
Geographic region				
East	15	Reference		
North	6	0.48 (0.22-1.05)^		
Northeast	2	0.87 (0.30-2.49)		
Northwest	5	0.74 (0.34-1.63)		
South Central	4	1.56 (0.71-3.43)		
Southwest	3	1.08 (0.44-2.61)		
Survey year (per decade increase)	35	1.27 (0.85-1.89)	10	0.50 (0.15-1.65)

*Note: * statistically significant (p<0.05); ^ p<0.1; $ the effect of sex was estimated based on studies that reported sex-specific cataract prevalence; the effect of geographic region on the prevalence of ARC was not conducted because of the deficiency of available information.*

Table S5. Full list of the included studies on cataract and cataract blindness prevalence in China (n=55)

Study ID	Reference
CA-01	Li S, Xu J, He M, Wu K, Munoz SR, Ellwein LB. A survey of blindness and cataract surgery in Doumen County, China. OPHTHALMOLOGY 1999;106(8):1602-8.
CA-02	Jia-liang Zhao, Rui-fang Sui, Li-jun Jia, et al. 赵家良, 睢瑞芳, 贾丽君, et al. Prevalence of cataract and surgical coverage among adults aged 50 or above in Shunyi district of Beijing, China (北京市顺义区白内障患病和手术状况的调查)[J]. Chin J Ophthalmol (中华眼科杂志). 2001(01):6-11.
CA-03	Xiang Tang. 唐香. Prevalence of cataract and surgical coverage among adults aged 50 or above in Luxi county of Yunnan province (云南省泸西县 50 岁及以上人群白内障患病情况及手术状况调查)[D]. Kunming Medical University (昆明医科大学), 2008.
CA-04	Xiao-yun Tang. 唐晓云. Investigation of the cataract epidemiology and surgery in Jiagedaqi District* (加格达奇区患白内障情况和手术状况的调查)[J]. Heilongjiang Medical Journal (黑龙江医学). 2002(02):148.
CA-05	Yun-xun Wang, Yan Zhao, Yong-mei Sun, et al. 王云旭, 赵艳, 孙永梅, et al. Establishment and implementation of cataract blindness prevention and treatment in Mongolian* (蒙古族白内障防盲治盲三级网站建立与实施)[J]. Ophthalmol CHN (眼科). 2003(03):160-1.
CA-06	Zhi-qing Li, Xiu-juan Zhang, Hui-min Sun, et al. 李志清, 张秀娟, 孙慧敏, et al. Investigation of cataract prevalence in people aged 40 years and above in Sangzi village, Ji County, Tianjin* (天津蓟县桑梓村 40 岁及以上人群白内障患病率调查)[J]. Chin J Pract Ophthalmol (中国实用眼科杂志). 2004(09):749-50.
CA-07	Lu Chen, Li-na Huang, Xiao-xia Li, et al. 陈璐, 黄丽娜, 李晓霞, 赖小兰, 曾平. Prevalence of cataract and surgical coverage among adults aged 50 or above in Baoan district of Shenzhen, China (深圳宝安区 50 岁及以上人群白内障患病情况及手术状况调查)[J]. International Journal of Ophthalmology (国际眼科杂志). 2004(05):919-21.
CA-08	Lin Ding, Xiu-rong Zhao, Xin Yang, et al. 丁琳, 赵秀蓉, 杨昕, 史韶华. Epidemiological investigation of cataract in Urumqi, Xinjiang* (新疆乌鲁木齐市白内障的流行病学调查)[J]. Xinjiang Medical Journal (新疆医学). 2005(01):40-2.
CA-09	Lin Li, Huai-jin Guan, Ji-bo Zhou, et al. 李琳, 管怀进, 周激波, et al. An epidemiological survey of cataract among adults aged 60 years and above in Xinchengqiao Blocks, Nantong (南通市新城桥街道 60 岁及以上人群白内障流行病学调查)[J]. Chin J Pract Ophthalmol (中国实用眼科杂志). 2006(07):752-7.

CA-10	Zhi-jian Li, Hao Cui, Ping Liu, et al. 李志坚, 崔浩, 刘平, 张丽琼, 李彬. Survey of cataract among the people aged 50 years and older in a rural area of Harbin (哈尔滨南部 50 岁及以上农村人口白内障的调查)[J]. International Journal of Ophthalmology (国际眼科杂志). 2007(05):1460-3.
CA-11	Xiao-bo Huang, Hai-dong Zou, Ning Wang, et al. 黄晓波, 邹海东, 王宁, et al. Epidemiological survey of cataract among the elder in Beixinjing Blocks, Shanghai (上海北新泾老人白内障流行病学调查)[J]. Int J Ophthalmol (国际眼科杂志). 2009(07):1321-4.
CA-12	Yan Zhou, Xue-ping Jia. 周燕, 贾雪平. An investigation of cataract prevalence in elderly in one community* (某社区老年人口白内障患病率调查)[J]. Guide of China Medicine (中国医药指南). 2011(16):136-7.
CA-13	Bin Tang, Zhi Li, Yi Luo, et al. 唐斌, 李治, 罗羿, et al. The survey comparisons on the epidemiology of cataracts among the old aged 50 and above in the urban and rural areas in Jiangbei district of Chongqing (重庆市江北区城乡 50 岁及以上人群白内障的流行病学调查)[J]. Chongqing Medicine (重庆医学). 2011(06):561-4.
CA-14	Ting-yu Xie, Yan Wang, Liang Gao, et al. 谢婷玉, 王燕, 高亮, et al. Prevalence of cataract and surgical coverage among Urger adults aged 40 or above in Kuche rural area of Xinjiang, China (新疆库车县维吾尔族农民白内障患病状况调查)[J]. Chin J Epidemiol (中华流行病学杂志). 2011;32(1):95-6.
CA-15	Ming-gui Zhao, Shi-hong Zhang, Le-xin Wang. 赵明贵, 张士红, 王乐新. An investigation report of cataract epidemiology in adults aged 50 years and above in rural Lanshan, Rizhao* (日照市岚山区农村 50 岁以上人群白内障流行病学调查报告)[J]. Chinese Community Doctors (中国社区医师（医学专业）). 2011(14):311-2.
CA-16	Hai-fang Zhang. 张海芳. An investigation on the prevalence of cataract and its surgical situation in Binhu district, Wuxi city * (无锡市滨湖区白内障患病和手术状况的调查) [J]. China Health Care and Nutrition (中国保健营养（下旬刊)). 2013;23(12):7654-5.
CA-17	Feng-rong Li, Jia-liang Zhao, Hong Lu, et al. 李凤荣, 赵家良, 陆宏, et al. Prevalence and surgery status of cataract among adults aged 50 years or above in the Shunyi district of Beijing: the China Nine-Province Survey (我国九省眼病调查中北京市顺义区 50 岁及以上人群白内障患病率和手术状况的调查)[J]. Chin J Ophthalmol (中华眼科杂志). 2012;48(3):211-8.
CA-18	Jing-xiang Bi. 毕经香. An epidemiological survey on cataract in different professions* (不同职业人群白内障患病情况的流行病学调查)[J]. Hebei Medicine (河北医学). 2012(09):1328-30.

CA-19	Huai-jin Guan, Hong Lu, Zhui Dai, et al. 管怀进, 陆宏, 戴追, et al. Prevalence and surgery status of cataract among adults aged 50 years or above in Qidong city of Jiangsu Province: the China Nine-Province Survey (我国九省眼病调查中江苏省启东市 50 岁及以上人群白内障患病率和手术状况的调查)[J]. Chin J Ophthalmol (中华眼科杂志). 2012;48(3):219-25.
CA-20	Rong Luo, Jia-liang Zhao, Jing-lin Yi, et al. 罗荣, 赵家良, 易敬林, et al. Province of blindness and low vision among adults aged 50 years or above in Ji'an county of Jiangxi province: the China Nine-Province Survey (我国九省眼病调查中江西省吉安县 50 岁及以上人群白内障患病率和手术状况的调查)[J]. Chin J Ophthalmol (中华眼科杂志). 2012;48(6):530-6.
CA-21A	Huai-jin Guan, Yong Yao, Cong-kai Liang, et al. 管怀进, 姚勇, 梁从凯, et al. Prevalence and surgical status of cataract among adults aged 50 years or above in rural Jiangsu Province (江苏省农村 50 岁及以上人群白内障患病率和手术状况调查)[J]. Natl Med J China (中华医学杂志). 2013;93(5):330-5.
CA-21B	Min Ji, Mei Yang, Rong-rong Zhu, et al. 季敏, 杨梅, 朱蓉嵘, et al. An investigation on reasons of bad prognosis after cataract surgery in people aged 50 years and above in rural Funing, Jiangsu* (江苏省阜宁县农村 50 岁及以上人群白内障术后视力恢复不良原因调查)[J].Med J of Communications (交通医学). 2015;29(5):457-9.
CA-22	Ning Cai, Miao-miao Chen, Yuan-sheng Yuan, et al. 蔡宁, 陈苗苗, 袁援生, 蔡山. Prevalence rate and surgery status of cataract in Luxi county of Yunnan Province (云南省泸西县白内障患病率及手术覆盖率调查)[J]. Journal of Kunming Medical University (昆明医科大学学报) 2013;34(5):74-8.
CA-23A	Wan-zhen Jiao. 焦万珍. Prevalence of visual impairment and blindness, prevalence and surgery status of cataract in rural older adults in Shandong province (山东省农村 50 岁及以上人群盲与视力损伤、白内障患病率及白内障手术状况的调查研究)[D]. Shandong University (山东大学), 2014.
CA-23B	Gui-min Wang. 王桂敏. Prevalence and surgery status of cataract among adults aged 50 years and above in Tengzhou city of Shandong province (山东省滕州市 50 岁及以上居民白内障患病率及手术服务利用研究)[D].Shandong University (山东大学), 2013.
CA-23C	Hong-ying Tang. 唐红迎. A prevalence study on blindness and vision impairment with moderate and severe degree due to cataract among rural residents aged 50 years and above in Juancheng county (鄄城县 50 岁及以上农村居民白内障致盲与中重度视力损伤现况研究)[D]. Shandong University (山东大学), 2012.
CA-24	Fang Tian, Bai-chao Chen, Yuan He, et al. 田芳, 任百超, 何媛, 贾俊, 刘慧峰, 裴金枝. An epidemiological survey of cataract among adults aged 50 years and above in rural, Shaanxi Province (陕西省农村 50 岁及以上人群白内障流行病学调查)[J]. Int Eye Sci (国际眼科杂志). 2014;14(4):629-32.

CA-25A	Wei Shen. 沈蔚. Prevalence and surgery status of cataract in adult rural Bai, Yi and Han nationality in Yunnan province (云南省白族、彝族和汉族农村人群白内障流行病学调查)[D]. Kunming Medical University (昆明医科大学), 2015.
CA-25B	Shen W, Yang Y, Yu M, et al. Prevalence and outcomes of cataract surgery in adult rural Chinese populations of the Bai nationality in Dali: the Yunnan minority eye study. PLOS ONE 2013;8(4):e60236.
CA-26	Tian-yu Wang. 王天宇. An epidemiological survey of cataract among adults aged 50 or above in Jing'an district in Shanghai (上海市静安区江宁街道 50 岁及以上人群白内障流行病学调查)[D].Shanghai Jiao Tong University School of Medicine (上海交通大学), 2015.
CA-27	Hui-fang Jiang, Yun Peng, Wen-quan Zhang, et al. 蒋惠芳, 彭云, 张文权, 孙伟, 俞正娟. Investigation of the prevalence of cataract and operation status in the elderly of Sanlin community (三林社区老年人白内障患病和手术状况调查)[J]. Chinese community doctors (中国社区医师). 2015(23):78-9.
CA-28	Xiao-xiang Long. 龙小香. An investigation of prevalence situation among cataract patients in Taizhou* (台州市白内障患者的患病情况调查)[J]. Journal of Traditional Chinese Medicine Management (中医药管理杂志). 2015(04):18-20.
CA-29	Yi Hu, Jian-ping Chen, Shu-xiang He, et al. 胡煜, 陈建萍, 贺书香, 林燕梅. Survey on status of cataract in Xiangdong district of Pingxiang Jiangxi in 2015 (2015 年江西萍乡湘东区各乡镇白内障情况的调查)[J]. China Modern Medicine (中国当代医药). 2016;23(7):164-6, 169.
CA-30	Hong-bing Cai. 蔡红兵. An epidemiological survey of cataract among people aged 50 and over in Luxi County of Yunnan Province (云南省泸西县 50 岁及以上人群白内障流行病学调查)[D]. Kunming Medical University (昆明医科大学), 2016.
CA-31	Jing Zhou, Yuan Yuan, Xu Zhang, et al. 周婧, 袁媛, 张徐, 杨梅, 管怀进. Prevalence and surgery status of cataract among adults aged 60 years or above in two villages of Nantong (2008 年江苏省南通市两个自然村 60 岁及以上人群白内障患病率和手术状况调查)[J]. Chin J Ophthalmol (中华眼科杂志). 2017;53(7):514-21.
CA-32	Hong-yan Yao, Hong-jian Zhou, Shan-jun Wu, et al. 姚红艳, 周宏健, 吴善君, 李黎. An epidemiological investigation of eye diseases in people aged 50 years and above in Beilun district, Ningbo city * (宁波市北仑区 50 岁以上人群眼病流行病学调查)[J]. Modern Practical Medicine (现代实用医学). 2012(5):544-5.
CA-33	Rui-fang Gao, Peng Li, Ai-min Sang, et al. 高瑞芳, 李鹏, 桑爱民, 谌绍林. Analysis on the prevalence of cataract of Hui and Han people in Xiji county (西吉县回汉族 50 岁以上人群白内障患病率分析)[J]. Modern Prevention Medicine (现代预防医学). 2012(02):342-4.
CA-34	Jian-qiong Geng. 耿剑琼. Analysis of prevalence and related factors of cataract among residents aged 50 years and above in Wushan county (武山县 50 岁及以上人群白内障患病率及相关因素分析)[D].Lanzhou University (兰州大学). 2017.

CA-35	Lan Luan, Yong Yao, Dong-hong Fu, et al. 栾兰, 姚勇, 傅东红, et al. Survey of the cataract prevalence and surgical coverage rate among 50 or above in Wuxi city (无锡市 50 岁及以上人群白内障患病率和手术情况调查)[J]. Chin J Exp Ophthalmol (中华实验眼科杂志). 2014;32(6):551-5.
CA-36	Zhao J, Ellwein LB, Cui H, et al. Prevalence and outcomes of cataract surgery in rural China the China nine-province survey. OPHTHALMOLOGY 2010;117(11):2120-8.
CA-37	Yi-zhou Fu, Huan-ran Chen, Zhong-ming Wang, et al. 伏奕舟, 陈焕然, 王仲明, 洪仲思. A survey of 5248 cases for eye diseases screening in Zhuhai (珠海市 5248 例体检者眼病患病率调查分析)[J]. Journal of Chinese Modern Ophthalmology (中华现代眼科学杂志). 2005(4).
CA-38	Ping Xu.徐萍. The situation and related factors of cataract and glaucoma in 729 university staffs * (高校教职工 729 名白内障、青光眼患病情况及相关因素分析)[J]. Chin J School Health (中国学校卫生) 2003(4).
CA-39	Su-feng Wu, Jing-xin Guo, You Lai. 吴素锋, 郭景新, 赖友. An investigation and analysis of the prevalence of cataract and surgeries in people aged 50 years and above in Yangdong area* (阳东地区 50 岁白内障患病及手术状况的调查与分析)[J]. China Medical Engineering (中国医学工程). 2015 (11):52, 55.
ARC-1A	Yang-sheng Peng, Ai-yi Zhou, Li Chen, et al. 彭秧生, 周爱意, 陈莉, 何媛, 任百超. Prevalence of age related cataract and blindness in rural areas of Shaanxi province (陕西省农村 50 岁以上人群白内障和盲的患病率调查)[J]. International Journal of Ophthalmology (国际眼科杂志). 2007;7(1):220-3.
ARC-1B	Yan-long Quan, Jian-gang Yang, Bai-chao Ren. 权彦龙, 杨建刚, 任百超. Epidemic survey for cataract in Yang county, Shaanxi Province (陕西省洋县白内障的流行病学调查)[D]. International Journal of Ophthalmology (国际眼科杂志). 2006;6(6):1464-7.
ARC-2	Jie Feng, Yu-jing Wan, Guo-hui Yang, et al. 冯洁, 万玉景, 杨国慧, et al. Contrast observation of cataract and surgical coverage in rural and urban defined population in Jining (社区与农村白内障患病及手术状况的对比观察)[J]. China Clin Prac Med (中国临床实用医学). 2008;2(5):36-7.
ARC-3	Jin-song Bai, Pei-lin An, Yu-ling Liu, et al. 柏劲松, 安培林, 刘玉玲, 付瑜. An investigation of cataract prevalence among adults aged 55 years and above in one rural area of Beijing* (北京市某区农村≥55 岁人群白内障患病情况调查)[J]. Guide of China Medicine (中国医药指南). 2012(17):113-4.
ARC-4	Wei Xiang, Feng Gao, Xun-lun Sheng, et al. 向伟, 高峰, 盛迅伦, et al. Prevalence investigation of age related cataract among population aged 50 years or elder in Tongxin of Ningxia (宁夏同心县 50 岁及以上人群年龄相关性白内障患病率调查)[J]. Ningxia Med J (宁夏医学杂志). 2015;37(5):405-7.

ARC-5	Hong Zheng, Pu-lin Yu, Yi-shu Hong, et al. 郑宏, 于普林, 洪依舒, 段春波, 杨泽, 高芳坤. A survey of the current status and distribution of cataract in the elderly (我国城乡老年人白内障的患病情况调查)[J]. Chin J Epidemiol (中华流行病学杂志). 2001(06):52-4.
ARC-6	Wei Cui, Zhi-ying Liu, Gui-bin Yu. 崔巍, 刘志英, 于桂斌. Survey of cataract rate and surgical coverage in agricultural and pastoral area of Keshenketeng in Inner Mongolia (内蒙古赤峰市克什克腾旗农牧区白内障患病率及手术覆盖率调查)[J]. Chin J Exp Ophthalmol (中华实验眼科杂志). 2012;30(5):462-6.
ARC-7	Hua Zeng, Yu-juan Zhan. 曾华, 詹玉娟. An investigation on age-related cataract in Haikougang, Haikou city * (海口市海口港区年龄相关性白内障发病状况调查)[J]. Hainan Medical Journal (海南医学). 2011(16):131-3.
ARC-8	Sheng Y, He F, Lin JF, Shen W, Qiu YW. Tea and Risk of Age-Related Cataracts: A Cross-Sectional Study in Zhejiang Province, China. J EPIDEMIOL 2016;26(11):587-92.
ARC-9	Wen-jie Liu. 刘文洁. Epidemiological survey of cataract among adults aged 40 or above in Gongshan County of Yunnan province (云南省怒江州贡山县 40 岁及以上人群白内障的流行病学调查)[D]. Tianjin Medical University (天津医科大学), 2009.
ARC-10	Li Chang, Li-juan Zhang, Jian Zhao, et al. 常莉, 张丽娟, 赵健, 赵媚鲜, 饶华祥. A survey of age-related cataract epidemiology in Taiyuan city (太原市年龄相关性白内障的流行病学调查)[J]. Proceeding of Clinical Medicine (临床医药实践). 2009(16):420-2.
ARC-11	Yong Yue. 岳镛. Investigation analysis of cataract among retired personnel of southwest university for nationalities (西南民族学院退离休职工白内障的调查)[J]. Journal of Southwest University for Nationalities. Natural Science Edition (西南民族学院学报(自然科学版)). 2001(03):378-80.

Note: The Chinese publication list employed the journals' official English names or abbreviations, English titles were obtained from journals or literature databases (CNKI, Wanfang and CBM). Where official English translation of journal names is not available, a pinyin title is adopted; where the English translation of titles is not available, I translated the titles, labelled with "" and marked as green.*

Table S6. Detailed characteristics of the included studies on cataract and cataract blindness prevalence in China (n=55)

Study ID	Study	Province	Region	Sex	Setting	Age range	Sampling	Study Year	Is ARC?	Sample size	Cataract	Cataract blindness (BCVA<0.05)	Cataract blindness (BCVA<0.1)	Cataract blindness (PVA<0.1)
**CA-01**	Li SZ et al. 1999	Guangdong	South Central China	Both	Rural	50+	Random cluster sampling	1997	Unclear	5288	-	-	-	206
**CA-02**	Zhao JL, et al. 2001	Beijing	North China	Both	Mixed	50+	Random cluster sampling	1996	Unclear	5084	1185	83	-	113
**CA-03**	Tang X 2008	Yunnan	Southwest China	Both	Mixed	50+	Random cluster sampling	2007	Unclear	5151	1154	-	-	-
**CA-04**	Tang XY 2002	Heilongjiang	Northeast China	Both	Urban	50+	Random cluster sampling	1999*	Unclear	3508	737	58	-	-
**CA-05**	Wang YX et al. 2003	Inner Mongolia	North China	Both	Rural	40+	Stratified random cluster sampling	2002	Unclear	4346	443	-	-	-
**CA-06**	Li ZQ et al. 2004	Tianjin	North China	Both	Rural	40+	Cluster sampling	2003	Unclear	1776	294	-	-	-
**CA-07**	Chen L et al. 2004	Guangdong	South Central China	Both	Urban	50+	Random cluster sampling	2002	Unclear	3428	1016	-	-	-
**CA-08**	Ding L et al. 2005	Xinjiang	Northwest China	Mixed	Urban	50+	Random cluster sampling	2002	Unclear	2055	714	-	-	-
**CA-09**	Li L et al. 2006	Jiangsu	East China	Both	Urban	60+	Random cluster sampling	2003	Unclear	3040	-	65	-	75
**CA-10**	Li ZJ et al. 2007	Heilongjiang	Northeast China	Both	Rural	50-96	Random cluster sampling	2007	Unclear	5058	1529	-	-	-
**CAT11**	Huang XB et al. 2009	Shanghai	East China	Both	Urban	60+	Random cluster sampling	2008	Unclear	3851	-	-	-	149

**CA-12**	Zhou Y et al. 2011	Henan	South Central China	Both	Urban	60+	Cluster sampling	2007	Unclear	3620	2499	-	-	-
**CA-13**	Tang B et al. 2011	Chongqing	Southwest China	Both	Both	50+	Cluster sampling	2007	Unclear	4587	-	188	-	323
**CA-14**	Xie TY et al. 2011	Xinjiang	Northwest China	Both	Rural	40-100	Random cluster sampling	2009	Unclear	4191	750	147	-	-
**CA-15**	Zhao MG et al. 2011	Shandong	East China	Both	Rural	50+	Cluster sampling	2007	Unclear	15720	4410	-	-	-
**CA-16**	Zhang HF 2013	Jiangsu	East China	Both	Urban	50-83	Random cluster sampling	2012	Unclear	4907	776	-	-	-
**CA-17**	Li FR et al. 2012	Beijing	North China	Both	Mixed	50+	Random cluster sampling	2006	Unclear	5118	797	-	135	-
**CA-18**	Bi JX 2012	Hebei	North China	Mixed	Mixed	40+	Cluster sampling	2010	Unclear	22565	6542	-	-	-
**CA-19**	Guan HJ et al. 2012	Jiangsu	East China	Both	Mixed	50+	Random cluster sampling	2006	Unclear	5141	1098	-	108	-
**CA-20**	Luo R et al. 2012	Jiangxi	East China	Both	Mixed	50+	Random cluster sampling	2006	Unclear	5010	1158	-	192	-
**CA-21A**	Guan HJ et al. 2013	Jiangsu	East China	Both	Rural	50+	Random cluster sampling	2010	Unclear	6106	720	-	113	-
**CA-21B**	J M et al. 2015	Jiangsu	East China	Both	Rural	50+	Random cluster sampling	2010	Unclear	5947	1488	-	120	-
**CA-22**	Cai N et al. 2013	Yunnan	Southwest China	Both	Mixed	50+	Random cluster sampling	2012	Unclear	5151	1154	-	-	-
**CA-23A**	Jiao WZ 2014	Shandong	East China	Both	Rural	50-101	Random cluster sampling	2008	Unclear	17816	4938	-	378	-
**CA-23B**	Wang GM 2013	Shandong	East China	Both	Rural	50+	Random cluster sampling	2008	Unclear	4916	1308	-	97	-
**CA-23C**	Tang HY 2012	Shandong	East China	Both	Rural	50+	Random cluster sampling	2008	Unclear	4866	1937	-	-	-
**CA-24**	Tian F et al. 2014	Shaanxi	Northwest China	Both	Rural	50-92	Stratified random cluster sampling	2011	Unclear	1912	701	-	-	-
**CA-25A**	Shen W 2015	Yunnan	Southwest China	Both	Rural	50-96	Random cluster sampling	2010	Unclear	6546	3172	-	-	518

**CA-25B**	Shen W et al. 2013	Yunnan	Southwest China	Both	Rural	50-96	Random cluster sampling	2010	Unclear	2133	-	-	-	144
**CA-26**	Wang TY 2015	Shanghai	East China	Both	Urban	50+	Random sampling	2013	Unclear	2044	588	-	-	65
**CA-27**	Jiang HF et al. 2015	Shanghai	East China	Both	Urban	65+	Cluster sampling	2014	Unclear	13101	5369	-	-	481
**CA-28**	Long XX 2015	Zhejiang	East China	Both	Both	60-85	Stratified cluster s ampling	2011	Unclear	1452	641	-	-	-
**CA-29**	Hu Y et al. 2016	Jiangxi	East China	Mixed	Mixed	50-90	Cluster sampling	2015	Unclear	4674	1483	-	-	-
**CA-30**	Cai HB 2016	Yunnan	Southwest China	Both	Mixed	50+	Random cluster sampling	2014	Unclear	5592	-	-	-	436
**CA-31**	Zhou J et al. 2017	Jiangsu	East China	Both	Rural	60-89	Cluster sampling	2008	Unclear	1305	-	-	42	-
**CA-32**	Yao HY et al. 2012	Zhejiang	East China	Both	Mixed	51-97	Random cluster sampling	2011	Unclear	1805	1084	-	-	-
**CA-33**	Gao RF et al. 2012	Ningxia	Northwest China	Both	Mixed	50-88	Cluster sampling	2007	Unclear	75197	5983	-	-	-
**CA-34**	Geng JQ 2017	Gansu	Northwest China	Both	Both	50-92	Stratified random cluster sampling	2014*	Unclear	1576	617	-	-	-
**CA-35**	Luan L et al. 2014	Jiangsu	East China	Both	Urban	50+	Random cluster sampling	2010	Unclear	6150	1785	112	-	-
**CA-36**	Zhao JL et al. 2010	Guangdong/Heilongjiang/Hebei/Xinjiang/Chongqing/Yunnan	North China, Northeast China, South Central China, Southwest China and Northwest China	Both	Mixed	50+	Random cluster sampling	2006	Unclear	30478	-	-	1324	-

**CA-37**	Fu YZ et al. 2005	Guangdong	South Central China	Both	Urban	50+	General health screening	2004	Unclear	808	140	-	-	-
**CA-38**	Xu P 2003	Beijing	North China	Mixed	Urban	40+	General health screening	2002	Unclear	634	124	-	-	-
**CA-39**	Wu SF et al. 2015	Guangdong	South Central China	Both	Urban	50+	General health screening	2014	Unclear	6870	2900	-	-	-
**ARC-1A**	Peng YS et al. 2007	Shaanxi	Northwest China	Both	Rural	50+	Stratified random cluster sampling	2003	Yes	1762	655	-	-	-
**ARC-1B**	Quan YL et al. 2006	Shaanxi	Northwest China	Both	Rural	50+	Stratified random cluster sampling	2003	Yes	1536	135	-	-	-
**ARC-2**	Feng J et al. 2008	Shandong	East China	Both	Both	50-100	Cluster sampling	2005*	Yes	2084	285	-	-	-
**ARC-3**	Bai JS et al. 2012	Beijing	North China	Both	Rural	55-109	Cluster sampling	2008	Yes	80673	2953	-	-	-
**ARC-4**	Xiang W et al. 2015	Ningxia	Northwest China	Both	Rural	50+	Random cluster sampling	2014	Yes	4812	1682	-	-	-
**ARC-5**	Zheng H et al. 2001	Beijing, Shanghai,Guangdong, Sichuan, Shaanxi, Liaoning	North China, Northeast China, East China, South Central China, Southwest China and Northwest China	Both	Mixed	60+	Stratified cluster s ampling	1997	Yes	8252	3865	-	-	-
**ARC-6**	Cui W et al. 2012	Inner Mongolia	North China	Both	Rural	50-92	Random cluster sampling	2010	Yes	3826	-	128	-	233

**ARC-7**	Zeng H et al. 2011	Hainan	South Central China	Both	Urban	60+	Cluster sampling	2009	Yes	1024	509	-	-	-
**ARC-8**	Sheng Y et al. 2016	Zhejiang	East China	Both	Mixed	60+	Random cluster sampling	2014	Yes	9343	409	-	-	-
**ARC-9**	Liu WJ 2009	Yunnan	Southwest China	Both	Rural	40+	Random cluster sampling	2008	Yes	1117	-	14	-	40
**ARC-10**	Chang L, et al. 2009	Shanxi	North China	Both	Urban	50+	Random health screening	2005	Yes	5216	804	-	-	-
**ARC-11**	Yue Y 2001	Sichuan	Southwest China	Both	Urban	50+	Health screening	1998*	Yes	423	74	-	-	-

Note: "-" represents unavailable data; "" indicates studies whose survey year was imputed.*

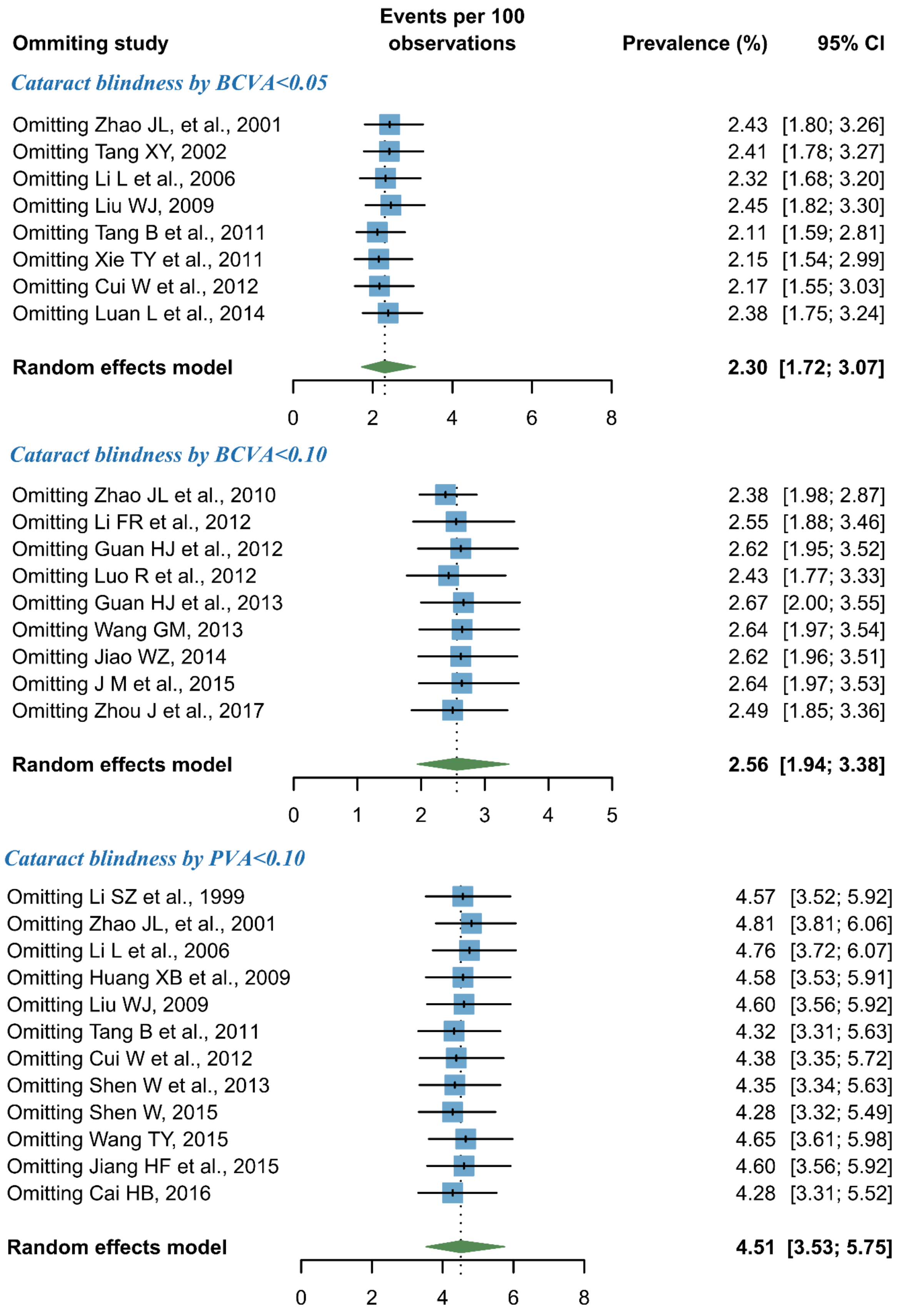

Figure S6. Leave-one-out sensitivity analysis of the influence of single study on the pooled prevalence of cataract blindness in China

Cataract blindness by BCVA<0.05

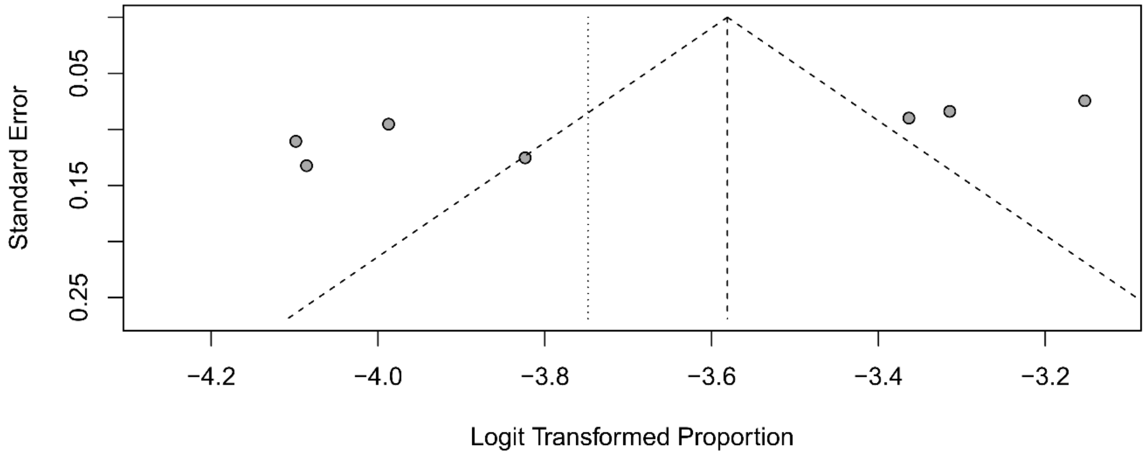

Cataract blindness by BCVA<0.10

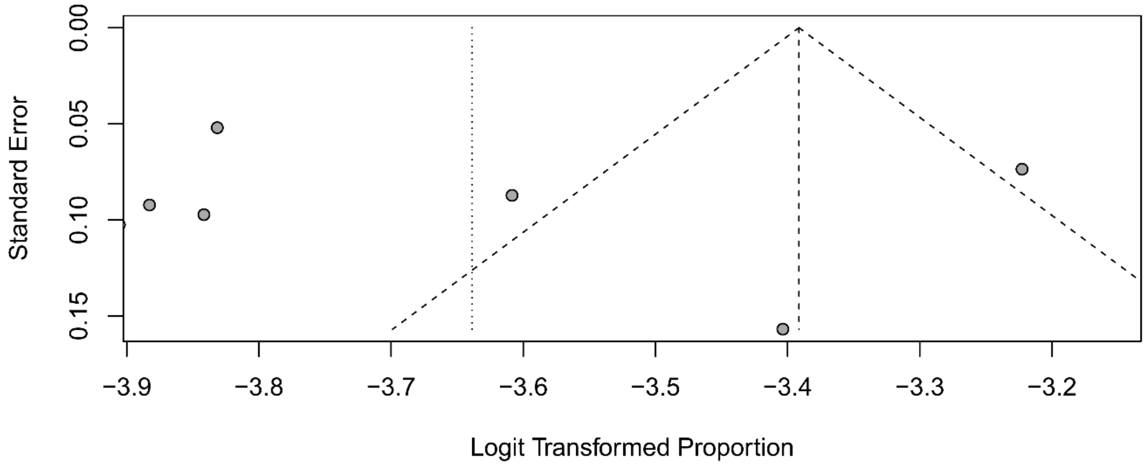

Cataract blindness by PVA<0.10

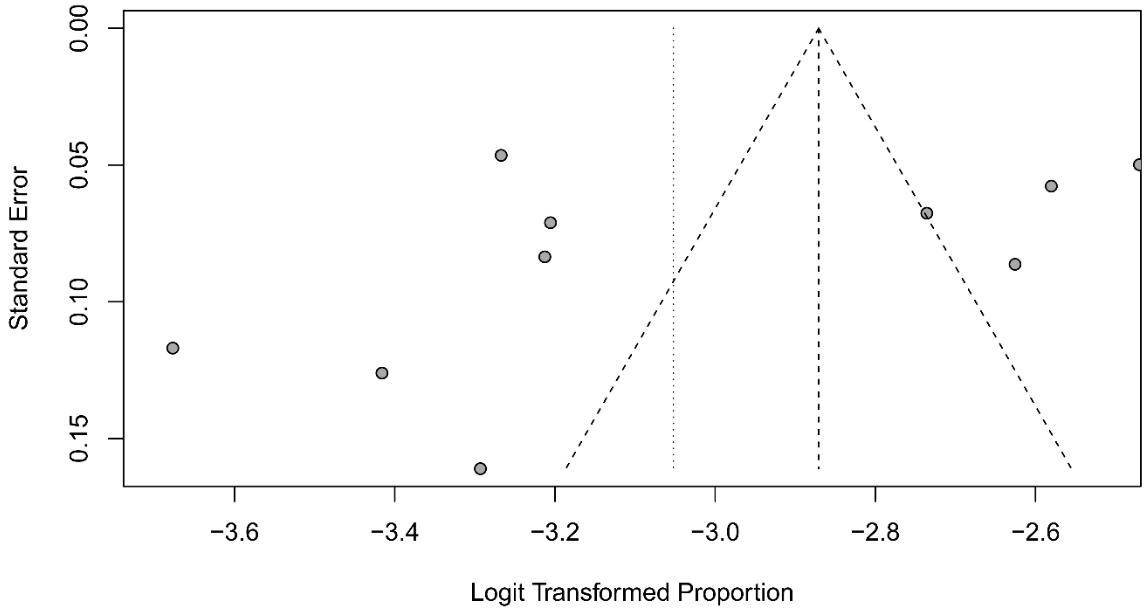

Figure S7. Funnel plot for the assessment of publication bias of the studies on the prevalence of cataract blindness in China

Table S7. Estimate and projection of the sex-specific number of people with any cataract and ARC in China from 1990 to 2050, by age group (million, 95% CI)

Age	Any cataract			ARC		
**1990**						
	**Male**	**Female**	**Overall**	**Male**	**Female**	**Overall**
45-49 years	1.71 (1.29-2.26)	1.93 (1.46-2.52)	3.64 (2.76-4.78)	0.83 (0.38-1.74)	1.08 (0.51-2.24)	1.91 (0.89-3.98)
50-54 years	2.45 (1.90-3.12)	2.68 (2.10-3.39)	5.12 (4.00-6.51)	1.26 (0.6-2.59)	1.61 (0.77-3.23)	2.88 (1.37-5.82)
55-59 years	3.24 (2.58-4.02)	3.59 (2.89-4.42)	6.83 (5.48-8.44)	1.80 (0.87-3.56)	2.33 (1.15-4.44)	4.14 (2.02-8.01)
60-64 years	3.67 (3.00-4.45)	4.13 (3.41-4.95)	7.80 (6.40-9.40)	2.22 (1.10-4.17)	2.90 (1.48-5.17)	5.12 (2.59-9.34)
65-69 years	3.72 (3.09-4.41)	4.45 (3.75-5.20)	8.17 (6.84-9.61)	2.46 (1.27-4.31)	3.38 (1.83-5.55)	5.84 (3.10-9.86)
70-74 years	3.70 (3.13-4.29)	4.61 (3.96-5.26)	8.31 (7.10-9.56)	2.67 (1.47-4.31)	3.77 (2.19-5.65)	6.45 (3.66-9.95)
75-79 years	2.52 (2.18-2.86)	3.67 (3.23-4.10)	6.20 (5.41-6.96)	1.99 (1.17-2.91)	3.21 (2.04-4.37)	5.20 (3.21-7.29)
80–84 years	1.20 (1.06-1.33)	2.20 (1.97-2.40)	3.40 (3.03-3.73)	1.02 (0.66-1.36)	2.02 (1.41-2.53)	3.04 (2.07-3.89)
85-89 years	0.39 (0.35-0.43)	0.89 (0.81-0.95)	1.28 (1.17-1.37)	0.35 (0.25-0.43)	0.85 (0.65-0.99)	1.20 (0.90-1.42)
Total (45-89 years)	22.60 (18.59-27.17)	28.15 (23.58-33.19)	50.75 (42.17-60.37)	14.61 (7.78-25.39)	21.17 (12.03-34.16)	35.77 (19.81-59.55)
**2000**						
**Age**	**Any cataract**			**ARC**		
	**Male**	**Female**	**Overall**	**Male**	**Female**	**Overall**
45-49 years	2.93 (2.21-3.86)	3.51 (2.66-4.59)	6.44 (4.88-8.45)	1.41 (0.66-2.97)	1.97 (0.93-4.08)	3.39 (1.59-7.05)
50-54 years	3.14 (2.44-4.01)	3.73 (2.92-4.73)	6.87 (5.37-8.74)	1.62 (0.77-3.33)	2.25 (1.08-4.50)	3.87 (1.85-7.82)
55-59 years	3.58 (2.86-4.45)	4.04 (3.25-4.97)	7.62 (6.11-9.42)	2.00 (0.96-3.95)	2.62 (1.29-4.99)	4.62 (2.26-8.94)
60-64 years	4.65 (3.80-5.64)	5.13 (4.23-6.15)	9.78 (8.03-11.79)	2.82 (1.40-5.29)	3.60 (1.85-6.42)	6.42 (3.24-11.71)
65-69 years	5.29 (4.40-6.27)	6.04 (5.09-7.06)	11.33 (9.49-13.33)	3.49 (1.81-6.13)	4.58 (2.48-7.53)	8.08 (4.29-13.66)
70-74 years	4.74 (4.01-5.50)	5.73 (4.93-6.55)	10.47 (8.94-12.05)	3.42 (1.88-5.52)	4.69 (2.73-7.02)	8.12 (4.61-12.54)
75-79 years	3.41 (2.94-3.87)	4.67 (4.11-5.22)	8.08 (7.05-9.08)	2.68 (1.59-3.93)	4.09 (2.59-5.56)	6.77 (4.18-9.49)
80–84 years	2.16 (1.90-2.39)	3.27 (2.93-3.57)	5.43 (4.83-5.96)	1.83 (1.18-2.44)	3.01 (2.10-3.76)	4.84 (3.28-6.20)
85-89 years	0.83 (0.75-0.90)	1.48 (1.36-1.58)	2.31 (2.10-2.48)	0.75 (0.53-0.92)	1.41 (1.08-1.65)	2.16 (1.61-2.56)
Total (45-89 years)	30.73 (25.31-36.89)	37.60 (31.48-44.41)	68.33 (56.79-81.29)	20.03 (10.77-34.47)	28.24 (16.12-45.52)	48.26 (26.89-79.99)
**2010**						
**Age**	**Any cataract**			**ARC**		
	**Male**	**Female**	**Overall**	**Male**	**Female**	**Overall**
45-49 years	3.46 (2.61-4.55)	4.15 (3.15-5.43)	7.61 (5.76-9.98)	1.67 (0.78-3.51)	2.33 (1.10-4.82)	4.00 (1.87-8.33)

50-54 years	4.21 (3.27-5.37)	5.00 (3.91-6.33)	9.20 (7.19-11.70)	2.17 (1.03-4.45)	3.01 (1.44-6.02)	5.19 (2.47-10.47)
55-59 years	6.26 (5.00-7.78)	7.42 (5.97-9.13)	13.69 (10.97-16.91)	3.49 (1.68-6.90)	4.82 (2.37-9.17)	8.31 (4.06-16.07)
60-64 years	6.17 (5.03-7.48)	7.28 (6.00-8.72)	13.45 (11.04-16.20)	3.73 (1.85-7.01)	5.11 (2.62-9.11)	8.84 (4.47-16.12)
65-69 years	6.15 (5.12-7.29)	6.96 (5.86-8.14)	13.11 (10.98-15.43)	4.06 (2.10-7.13)	5.29 (2.86-8.69)	9.35 (4.96-15.81)
70-74 years	6.47 (5.48-7.51)	7.39 (6.36-8.44)	13.86 (11.83-15.95)	4.68 (2.56-7.53)	6.05 (3.52-9.05)	10.72 (6.08-16.59)
75-79 years	5.34 (4.61-6.06)	6.73 (5.91-7.51)	12.07 (10.52-13.57)	4.21 (2.49-6.17)	5.88 (3.73-8.01)	10.09 (6.22-14.18)
80–84 years	3.13 (2.76-3.47)	4.45 (4.00-4.86)	7.58 (6.76-8.33)	2.66 (1.72-3.55)	4.10 (2.86-5.12)	6.76 (4.58-8.67)
85-89 years	1.33 (1.20-1.44)	2.17 (1.99-2.32)	3.50 (3.19-3.77)	1.20 (0.85-1.47)	2.07 (1.58-2.42)	3.27 (2.44-3.89)
Total (45-89 years)	42.52 (35.08-50.96)	51.56 (43.16-60.88)	94.07 (78.24-111.84)	27.86 (15.07-47.72)	38.67 (22.08-62.42)	66.54 (37.15-110.14)
**2015**						
**Age**	**Any cataract**			**ARC**		
	**Male**	**Female**	**Overall**	**Male**	**Female**	**Overall**
45-49 years	4.20 (3.17-5.53)	5.11 (3.87-6.68)	9.31 (7.05-12.21)	2.02 (0.94-4.26)	2.87 (1.35-5.94)	4.90 (2.29-10.20)
50-54 years	5.15 (4.00-6.57)	6.14 (4.81-7.78)	11.29 (8.81-14.35)	2.66 (1.26-5.45)	3.70 (1.77-7.40)	6.36 (3.03-12.85)
55-59 years	6.06 (4.84-7.53)	7.18 (5.78-8.82)	13.24 (10.61-16.35)	3.38 (1.63-6.68)	4.66 (2.30-8.87)	8.04 (3.93-15.54)
60-64 years	8.58 (7.00-10.40)	10.15 (8.37-12.16)	18.73 (15.37-22.56)	5.19 (2.58-9.75)	7.13 (3.65-12.71)	12.32 (6.23-22.46)
65-69 years	7.78 (6.47-9.23)	9.26 (7.80-10.82)	17.04 (14.27-20.05)	5.14 (2.66-9.01)	7.03 (3.80-11.55)	12.17 (6.46-20.56)
70-74 years	6.87 (5.81-7.97)	7.97 (6.86-9.11)	14.84 (12.67-17.07)	4.96 (2.72-8.00)	6.53 (3.79-9.77)	11.49 (6.51-17.77)
75-79 years	6.04 (5.21-6.85)	7.33 (6.44-8.18)	13.36 (11.65-15.03)	4.75 (2.81-6.97)	6.41 (4.06-8.72)	11.16 (6.87-15.69)
80–84 years	3.99 (3.52-4.43)	5.45 (4.89-5.95)	9.45 (8.41-10.38)	3.39 (2.19-4.53)	5.02 (3.50-6.27)	8.41 (5.69-10.80)
85-89 years	1.77 (1.60-1.92)	2.72 (2.49-2.91)	4.49 (4.09-4.83)	1.60 (1.13-1.96)	2.60 (1.98-3.03)	4.20 (3.12-4.99)
Total (45-89 years)	50.44 (41.63-60.43)	61.30 (51.31-72.41)	111.74 (92.94-132.84)	33.10 (17.93-56.61)	45.94 (26.21-74.25)	79.04 (44.14-130.85)
**2020**						
**Age**	**Any cataract**			**ARC**		
	**Male**	**Female**	**Overall**	**Male**	**Female**	**Overall**
45-49 years	4.04 (3.05-5.32)	4.84 (3.67-6.33)	8.88 (6.72-11.65)	2.87 (1.35-5.94)	2.72 (1.28-5.63)	4.67 (2.19-9.73)
50-54 years	6.26 (4.87-7.99)	7.57 (5.93-9.59)	13.83 (10.80-17.58)	3.70 (1.77-7.40)	4.57 (2.19-9.12)	7.80 (3.72-15.75)
55-59 years	7.44 (5.94-9.24)	8.83 (7.11-10.86)	16.27 (13.05-20.1)	4.66 (2.30-8.87)	5.74 (2.83-10.92)	9.88 (4.83-19.11)
60-64 years	8.35 (6.81-10.12)	9.85 (8.12-11.80)	18.20 (14.93-21.92)	7.13 (3.65-12.71)	6.91 (3.54-12.33)	11.97 (6.05-21.81)
65-69 years	10.93 (9.09-12.96)	12.99 (10.95-15.19)	23.92 (20.04-28.15)	7.03 (3.80-11.55)	9.87 (5.34-16.21)	17.09 (9.08-28.87)

70-74 years	8.83 (7.48-10.25)	10.73 (9.23-12.25)	19.55 (16.70-22.50)	6.53 (3.79-9.77)	8.78 (5.10-13.15)	15.16 (8.60-23.43)
75-79 years	6.57 (5.67-7.46)	8.06 (7.08-9.00)	14.63 (12.75-16.45)	6.41 (4.06-8.72)	7.05 (4.47-9.59)	12.22 (7.53-17.18)
80–84 years	4.67 (4.12-5.18)	6.12 (5.49-6.68)	10.79 (9.61-11.86)	5.02 (3.50-6.27)	5.63 (3.93-7.03)	9.60 (6.49-12.33)
85-89 years	2.36 (2.12-2.56)	3.48 (3.19-3.72)	5.84 (5.31-6.28)	2.60 (1.98-3.03)	3.32 (2.54-3.87)	5.45 (4.05-6.48)
Total (45-89 years)	59.45 (49.15-71.08)	72.47 (60.76-85.42)	131.92 (109.91-156.49)	45.94 (26.21-74.25)	54.59 (31.21-87.85)	93.83 (52.52-154.69)
**2030**						
**Age**	**Any cataract**			**ARC**		
	**Male**	**Female**	**Overall**	**Male**	**Female**	**Overall**
45-49 years	3.35 (2.53-4.40)	3.98 (3.02-5.21)	7.32 (5.55-9.61)	1.61 (0.75-3.39)	2.24 (1.05-4.63)	3.85 (1.80-8.02)
50-54 years	4.81 (3.74-6.14)	5.70 (4.46-7.22)	10.51 (8.20-13.35)	2.48 (1.17-5.09)	3.44 (1.65-6.86)	5.92 (2.82-11.95)
55-59 years	8.77 (7.00-10.89)	10.36 (8.34-12.74)	19.13 (15.34-23.63)	4.89 (2.36-9.66)	6.73 (3.31-12.80)	11.62 (5.67-22.46)
60-64 years	12.62 (10.30-15.30)	15.07 (12.43-18.05)	27.69 (22.73-33.35)	7.64 (3.79-14.34)	10.58 (5.42-18.86)	18.22 (9.21-33.20)
65-69 years	13.35 (11.11-15.84)	15.75 (13.27-18.41)	29.11 (24.38-34.25)	8.82 (4.57-15.47)	11.96 (6.47-19.66)	20.78 (11.04-35.13)
70-74 years	12.56 (10.63-14.57)	15.02 (12.92-17.15)	27.57 (23.55-31.72)	9.07 (4.98-14.62)	12.29 (7.15-18.40)	21.36 (12.12-33.02)
75-79 years	12.66 (10.93-14.37)	15.95 (14.01-17.81)	28.62 (24.95-32.18)	9.97 (5.89-14.62)	13.95 (8.84-18.98)	23.92 (14.74-33.61)
80–84 years	7.17 (6.32-7.95)	9.75 (8.75-10.64)	16.92 (15.07-18.59)	6.08 (3.93-8.12)	8.98 (6.26-11.21)	15.06 (10.19-19.33)
85-89 years	3.36 (3.03-3.64)	4.79 (4.39-5.12)	8.15 (7.41-8.76)	3.03 (2.15-3.72)	4.57 (3.49-5.32)	7.60 (5.64-9.04)
Total (45-89 years)	78.65 (65.58-93.11)	96.36 (81.58-112.35)	175.01 (147.16-205.46)	53.60 (29.60-89.04)	74.73 (43.64-116.73)	128.33 (73.24-205.78)
**2040**						
**Age**	**Any cataract**			**ARC**		
	**Male**	**Female**	**Overall**	**Male**	**Female**	**Overall**
45-49 years	3.64 (2.75-4.79)	4.10 (3.11-5.37)	7.74 (5.86-10.16)	1.75 (0.82-3.69)	2.31 (1.09-4.78)	4.06 (1.90-8.46)
50-54 years	6.57 (5.11-8.38)	7.58 (5.93-9.60)	14.15 (11.04-17.98)	3.39 (1.60-6.95)	4.57 (2.19-9.13)	7.96 (3.80-16.08)
55-59 years	7.31 (5.84-9.08)	8.55 (6.89-10.52)	15.87 (12.72-19.60)	4.08 (1.97-8.05)	5.55 (2.74-10.57)	9.63 (4.70-18.62)
60-64 years	9.82 (8.01-11.90)	11.42 (9.42-13.68)	21.24 (17.43-25.58)	5.94 (2.95-11.16)	8.02 (4.11-14.29)	13.96 (7.06-25.45)
65-69 years	16.13 (13.41-19.13)	18.74 (15.79-21.91)	34.87 (29.21-41.04)	10.66 (5.52-18.69)	14.23 (7.70-23.39)	24.89 (13.22-42.08)
70-74 years	19.82 (16.79-23.01)	23.60 (20.30-26.96)	43.42 (37.09-49.96)	14.32 (7.85-23.08)	19.32 (11.23-28.92)	33.64 (19.09-52.01)
75-79 years	16.63 (14.36-18.88)	20.27 (17.80-22.62)	36.90 (32.16-41.50)	13.10 (7.74-19.21)	17.72 (11.24-24.12)	30.82 (18.98-43.33)
80–84 years	11.35 (10.01-12.59)	14.74 (13.23-16.09)	26.09 (23.23-28.68)	9.64 (6.23-12.86)	13.57 (9.46-16.95)	23.21 (15.69-29.82)
85-89 years	7.45 (6.71-8.08)	10.67 (9.78-11.41)	18.12 (16.49-19.49)	6.71 (4.77-8.24)	10.19 (7.78-11.87)	16.90 (12.55-20.11)

Total (45-89 years)	98.72 (82.98-115.84)	119.69 (102.26-138.17)	218.41 (185.24-254.01)	69.59 (39.44-111.94)	95.49 (57.53-144.03)	165.08 (96.98-255.97)
**2050**						
**Age**	**Any cataract**			**ARC**		
	**Male**	**Female**	**Overall**	**Male**	**Female**	**Overall**
45-49 years	2.61 (1.97-3.44)	2.83 (2.14-3.70)	5.44 (4.12-7.13)	1.26 (0.59-2.65)	1.59 (0.75-3.29)	2.85 (1.33-5.93)
50-54 years	4.08 (3.18-5.21)	4.47 (3.50-5.66)	8.55 (6.67-10.87)	2.11 (1.00-4.32)	2.69 (1.29-5.38)	4.80 (2.29-9.70)
55-59 years	8.01 (6.39-9.94)	8.86 (7.13-10.89)	16.86 (13.52-20.83)	4.46 (2.15-8.82)	5.75 (2.83-10.94)	10.21 (4.99-19.76)
60-64 years	13.57 (11.07-16.45)	15.29 (12.61-18.32)	28.86 (23.68-34.77)	8.21 (4.08-15.42)	10.73 (5.50-19.14)	18.94 (9.58-34.56)
65-69 years	13.74 (11.43-16.30)	15.66 (13.20-18.31)	29.41 (24.63-34.61)	9.08 (4.70-15.92)	11.89 (6.43-19.54)	20.97 (11.14-35.47)
70-74 years	16.04 (13.58-18.62)	18.28 (15.73-20.88)	34.32 (29.31-39.50)	11.59 (6.36-18.68)	14.97 (8.70-22.40)	26.56 (15.06-41.09)
75-79 years	21.50 (18.56-24.40)	25.05 (22.01-27.96)	46.55 (40.56-52.36)	16.93 (10.00-24.82)	21.90 (13.89-29.82)	38.83 (23.89-54.64)
80–84 years	19.90 (17.54-22.08)	24.67 (22.14-26.93)	44.58 (39.68-49.01)	16.90 (10.92-22.56)	22.72 (15.83-28.37)	39.61 (26.75-50.93)
85-89 years	11.32 (10.19-12.27)	14.95 (13.70-15.99)	26.27 (23.89-28.27)	10.20 (7.24-12.53)	14.28 (10.91-16.63)	24.48 (18.15-29.16)
Total (45-89 years)	110.76 (93.92-128.70)	130.07 (112.15-148.65)	240.83 (206.07-277.35)	80.73 (47.04-125.71)	106.53 (66.13-155.52)	187.26 (113.17-281.23)

REFERENCES - ONLINE SUPPLEMENTARY DOCUMENT

1. Higgins J, Thompson SG. Quantifying heterogeneity in a meta - analysis. Stat Med. 2002;21:1539-1558.
2. Higgins JP, Thompson SG, Deeks JJ, Altman DG. Measuring inconsistency in meta-analyses. Bmj. 2003;327:557-560.
